# Effect of different concentrations of heparin-locking solution for central venous catheters in hemodialysis patients: A systematic review and meta-analysis

**DOI:** 10.1371/journal.pone.0320207

**Published:** 2025-03-25

**Authors:** Lin Wang, Hui Wang, Yuxi Wang, Yuwen Wang, Yuxiu Liu

**Affiliations:** 1 School of Nursing, Shandong Second Medical University, Weifang, China; 2 Department of Burns and Wound Repair, Weifang People’s Hospital, Weifang, China; Ataturk University Faculty of Medicine, TÜRKIYE

## Abstract

**Background:**

The effect of different concentrations of heparin-locking solution for central venous catheters (CVC) in hemodialysis patients is varied. Regarding the optimal concentration of heparin-locking solution, there is no definitive consensus based on evidence.

**Objective:**

To investigate the efficacy and safety of different concentrations of heparin for CVC locking in hemodialysis patients.

**Methods:**

We searched PubMed, Embase, Web of science, the Cochrane Library and Clinical Trial Database (clinicaltrials.gov) from establishment of each database to September 19, 2024 for randomized control trials (RCTs), non-randomized control trials (NRCTs) and cohort study of heparin-locking solution for CVC in hemodialysis patients aged ≥ 18 years. Outcome data regarding catheter occlusion, hemodialysis blood flow, bleeding-related complications, catheter-related infection, and catheter retention time were extracted and pooled from selected studies. The quality of included RCTS, cohort studies, and NRCT were assessed using bias risk tools recommended in the Cochrane Handbook. A fixed-effect model was used to calculate pooled odds ratios or mean differences with 95% confidence intervals.

**Results:**

Three RCTs, three cohort studies and one NRCT involving 946 hemodialysis patients were included. Heparin-locking solution of 1000 U/ml reduces the occurrence of bleeding related complications (OR =  0.20, 95%CI: 0.05-0.79, P = 0.02) compared to 5000 U/ml and 10000 U/ml. No significant difference was observed in hemodialysis blood flow (MD = -6.95, 95% CI: -14.41- 0.51, P = 0.07), catheter occlusion (OR =  0.89, 95% CI =  0.60-1.33, P = 0.58), catheter retention time (MD = -0.16, 95% CI: -1.98 – 1.67, P = 0.87), and catheter-related infection (OR = 0.60, 95%CI: 0.31-1.19, P = 0.14).

**Conclusion:**

Our findings suggest that a heparin-locking solution at a concentration of 1000 U/ml for central venous catheters (CVC) in hemodialysis patients may reduce the incidence of bleeding-related complications, without impacting the rates of catheter occlusion, hemodialysis blood flow, catheter retention time, or catheter-related infections. However, due to the limited number of included studies, high-quality randomized controlled trials and cohort studies with long-term follow-up are warranted to further validate these results.

## Introduction

Hemodialysis (HD) is a renal replacement therapy (RRT) that purify the patient’s blood through cardiopulmonary bypass with the vascular access to remove accumulated metabolic waste [1, 2]. About 89% of dialysis patients receive hemodialysis worldwide [3]. The common vascular access in clinical hemodialysis includes arteriovenous graft fistula, autogenous arteriovenous fistula and central venous catheters (CVC) [4]. However, it takes weeks or even months for autologous arteriovenous fistula and arteriovenous graft fistula from establishment to full functional maturity, during which time a CVC is required as temporary vascular access. In addition, central venous catheters should be the only vascular access option for some patients who are not suitable for arteriovenous fistula or artificial arteriovenous transplantation. Nevertheless, catheter-related complications, such as bleeding, infection, and catheter occlusion, are significantly correlated with morbidity and mortality in HD patients [5]. One cohort study [6] of 1041 patients treated with maintenance HD reported a 9%, 15%, and 2% risk of catheter-related bacteremia, functional impairment, and central stenosis, respectively, one year after retention CVC. Maintaining good function of the catheter is a prerequisite for the success of hemodialysis treatment. The use of anticoagulants to lock the catheter lumen after HD therapy is a common nursing practice to reduce the occurrence of complications and prolong catheter use [7].

Heparin is a kind of mucopolysaccharide with sulfuric acid chemical group. Negatively charged heparin changes the configuration of AT-III by binding to positively charged arginine on the antithrombin III (AT-III) molecule. AT-III molecules exposing the arginine active site bind to serine-containing coagulation factors, rendering the coagulation factors inactive and achieving the effect of anticoagulation [8]. Intermittent flushing and locking of the catheter with heparin-locking solution has become the most commonly used procedure during hemodialysis treatment.

Although there have been some studies contrasting the efficacy of different concentrations of heparin-locking solution, there is no uniform consensus regarding the concentration of heparin-locking solution [9]. The KDOQI guidelines recommend the use of citrate or heparin for catheter locking, based on clinical judgment, to prevent central venous catheter (CVC) dysfunction. However, the guidelines do not provide specific recommendations regarding the concentration of heparin to be used [4]. Differences have been noted in the heparin-locking solution concentrations between different dialysis centers, ranging from 1,000 to 10,000 U/ ml. It is noteworthy that numerous studies have shown that higher heparin concentrations may increase the systemic activated partial thromboplastin time (APTT), thereby increasing the risk of bleeding and infection, which may be life-threatening of patients. Therefore, it has been suggested to minimize the exposure of dialysis patients to unnecessary and inappropriate anticoagulation. Several studies have explored this issue, generally indicating that a heparin concentration of 5000 U/ml serves as a cut-off point for comparing the locking efficacy of various heparin concentrations [10–13]. A meta-analysis conducted by Han et al. [10] found that low concentration (< 5000 U/ml) heparin-locking solution could reduce the occurrence of catheter-related infection and bleeding-related complications without influencing the catheter retention time or the incidence of catheter thrombosis/occlusion or catheter dysfunction. Renaud et al. [12] retrospectively observed 238 patients with low-risk bleeding after CVC implantation and found that catheter-related infection rate, immediate catheter-related bleeding and malfunction were not influenced by heparin-locking solution concentrations (≤5000 U/ml), but this needs to be further corroborated in higher risk patients of bleeding and malfunction. Tan et al’ s [11] meta-analysis found that no significant difference between 1000 U/ml and 5000 U/ml heparin-locking solution in efficacy and safety. There is still no consensus on the optimal heparin-locking solution concentration with respect to antithrombotic effect and safety.

Based on the inconsistent evidence of heparin-locking solution, this study aimed to compare the efficacy and safety of high and low concentrations of heparin-locking solution in hemodialysis patients, using 5000 U/ml as the cutoff point.

## Methods

### Search strategy and eligibility criteria

This study was guided by PRISMA criteria for systematic reviews and meta-analysis [14]. The PubMed, Embase, Web of Science, the Cochrane Library and Clinical Trial Database (clinicaltrials.gov) were searched for publications, from establishment of each database to September 19, 2024. The search terms consisted of three areas: 1) Central Venous Catheters; 2) Hemodialysis; 3) Heparin. The subject terms used in the search included “Central Venous Catheters,” “Renal dialysis,” and “Heparin,” while free terms comprised “Venous Catheters, Central,” “Central Venous Catheter,” “Hemodialysis,” “Extracorporeal Dialyses,” “Unfractionated Heparin,” and “Heparin Sodium.” For a complete list of the used keywords, see S1 Table. Related articles published in English were recruited. Potentially relevant studies that may not have been retrieved in the above databases were identified by screening the included articles from previous systematic reviews on heparin-locking solution for CVC in hemodialysis patients. This is a review article. As this study was based on published data, there was no need to re-solicit participants’ consent.

Studies were considered eligible if they met the following criteria: 1) patients over 18 years of age with central venous catheters for hemodialysis as participants; 2) patients underwent two or more heparin-locking solution of different concentrations; 3) at least one of the following outcome indicators were reported: catheter occlusion, hemodialysis blood flow (mL/min), catheter retention time, catheter-related infection, bleeding-related complications; and 4) an RCT, NRCT or cohort design was used. Articles that were guidelines, systematic reviews, meta-analyses, and without complete research data were excluded.

### Data extraction

The information extracted included the following areas: first author’s name, year of publication, country, study type, catheter type, sample size, average age of patients, intervention concentrations, catheter location, follow-up period, and outcome indicators. Outcome indicators were divided into efficacy (includes catheter occlusion, hemodialysis blood flow and catheter retention time) and safety (bleeding-related complications and catheter-related infection). Studies with missing complete data were excluded when the authors were contacted without access to the original data.

### Assessment of risk of bias

The quality of included RCTS, cohort studies, and NRCT were assessed using bias risk tools recommended in the Cochrane Handbook. The tool used to evaluate the RCTs included seven dimensions: generation of random sequence, concealment of allocation scheme, blinding of participants and study subjects, blinding of outcome assessment, completeness of outcome data, selective outcome reporting and ‘other’ questions. Each domain could be categorized as low risk of bias, high risk of bias, or unclear risk of bias according to the judgement criteria. Newcastle-Ottawa Scale (NOS) [15] was used to assess the risk of bias in cohort studies from three dimensions: selection, comparability and outcome. The total scale score was 9, with scores of 7-9 considered high-quality studies, 4-6 moderate-quality studies, and less than 4 low-quality studies. ROBINS-I [16] was used to assess the risk of bias in NRCTs. It included seven evaluation domains, which were divided into three parts: pre-intervention, at-intervention and post-intervention: 1) pre-intervention (confounding bias and object selection bias); 2) at-intervention (bias in intervention classification); 3) post-intervention (bias away from established intervention, bias of missing data, bias of outcome measurement and bias of outcome selectivity reporting). Each evaluation domain consists of multiple signaling questions, totaling 34 signal questions. The risk of bias in each domain was evaluated by answering the relevant signaling questions, which was classified as low, moderate, serious, critical, or no information. The overall risk of bias was evaluated based on the results of all individual domain evaluations. The risk of bias was determined by two researchers independently. If there was debate about the evaluation, a third researcher would be invited to participate in the discussion.

### Statistical analysis

Review Manage (RevMan) version 5.4 software was used to combine the results of the various studies and assess the overall effect of different concentrations of heparin-locking solution for central venous catheters in HD patients. We used risk Odds Ratio (OR) with 95% confidence interval (CI) to compare any effect on dichotomous variables (i.e., incidence of catheter occlusion, bleeding-related complications and catheter-related infection). As for comparative results for continuous variables, such as the hemodialysis blood flow and catheter retention time, were compared with mean difference (MD). We used a random-effects model for both the goal of statistical inference and for consideration of heterogeneity among included studies. When the intention to generalize the results beyond the included studies (generalization inference), the random-effects model is the appropriate statistical model for meta-analysis. When the intention to apply the results only to the included studies (no generalizations), the fixed-effects model is the appropriate statistical model [17]. And although the same drug was used across trials (heparin), clear clinical and methodological heterogeneous in the study methods applied: (i.e., different follow-up times, different kinds of patients, different study designs etc.). The heterogeneity was quantified by *I*^*2*^ statistics. Thresholds for the interpretation of the *I*^2^ statistic can be misleading, since the importance of inconsistency depends on several factors. A rough guide to interpretation in the context of meta-analyses of randomized trials is as follows: ① 0% to 40%: might not be important; ② 30% to 60%: may represent moderate heterogeneity; ③ 50% to 90%: may represent substantial heterogeneity; ④ 75% to 100%: considerable heterogeneity [18]. Furthermore, the sensitivity analysis was conducted in studies with high heterogeneity by using the means of removing single study one by one to observe whether the heterogeneity was decreased. The overall effect of the test was determined by the size of the P value, and P <  0.05 was considered statistically significant.

## Results

### Search results

**Fig 1:**
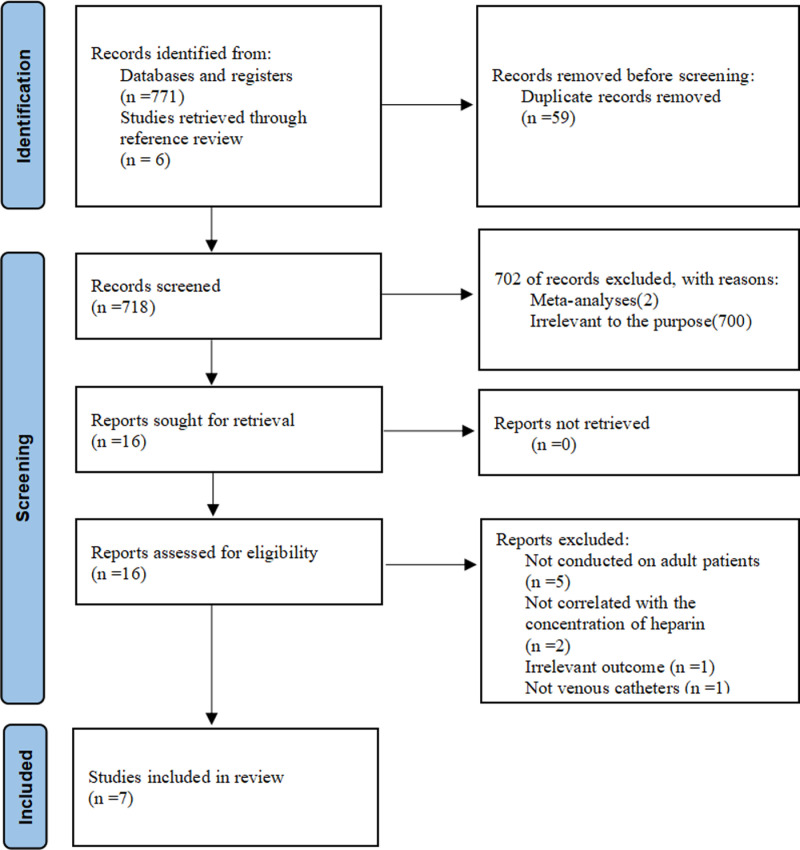
Flow chart of the study selection process.

After the preliminary systematic search, a total of 777 articles were retrieved, and 718 articles remained after eliminating the duplicate articles. A total of 16 articles were screened for full text, and 7 studies met the inclusion criteria. The specific literature screening process is shown in Fig 1.

### Study characteristics

Study characteristics are presented in Table 1. One NRCT [19], three cohort studies [12,20,21], and three RCT [13,22,23] were included. A total of 946 patients were involved in our study, among which Thomas et al.[19] had the largest sample size (263 cases), while Thomson et al.[22] only involved 28 patients. Four of these studies [12,13,20,21] focused on tunneled hemodialysis catheters, two [22, 23] on non-tunneled hemodialysis catheters, and one study [19] had no restrictions on catheter type. Catheter occlusion and catheter-related infection were reported in six [12,13,19,21–23] of seven the studies. Hemodialysis blood flow was reported in three studies [13,21,22]. Four studies [12,19,20,23] reported bleeding-related complications, and two studies [21,23] reported catheter retention times. The study of Thomas et al. [19] compared the effect of 1000U/ml and 10000U/ml heparin-locking solution, and other six studies compared 1000U/ml and 5000U/ml heparin-locking solution.

**Table 1:** Studies and Patients Characteristics in the metaanalysis.

Author	Country	Year	Catheter type	Study type	Patients (n)	Average age (years)	Interventions		Catheter location	Follow-up period	Outcome measure
Thomas et al.[19]	Canada	2007	tunneled or non-tunneled	NRCT	263 (130/133)	64.5	saline with 1000U/ml heparin	saline with 10000U/ml heparin	NR	6 months or 3 months	A、B、D
Yevzlin et al.[20]	USA	2007	tunneled	Cohort study	143 (91/52)	56	saline with 1000U/ml heparin	saline with 5000U/ml heparin	NR	NR	A
Maya et al.[21]	USA	2010	tunneled	Cohort study	105 (47/58)	54	saline with 1000U/ml heparin	saline with 5000U/ml heparin	right/left internal jugular vein	3 months	B、C、D、E
Hu et al.[23]	China	2011	non-tunneled	RCT	99 (48/51)	68	saline with 1000U/ml heparin	saline with 5000U/ml heparin	Jugular/ femoral vein	14 days or until catheter removal	A、B、C、D
Thomson et al.[22]	UK	2011	non-tunneled	RCT	28 (13/15)	68 (median)	saline with 1000U/ml heparin	saline with 5000U/ml heparin	NR	until catheter removal	B、D、E
Renaud et al.[12]	Singapore	2015	tunneled	Cohort study	208 (180/28)	60	saline with 1000U/ml heparin	saline with 5000U/ml heparin	right internal jugular	30days	A、B、D
Chu et al.[13]	Australia	2016	tunneled	RCT	100 (48/52)	62	saline with 1000U/ml heparin	saline with 5000U/ml heparin	right/left internal jugular/femoral vein	90days	B、D、E

RCT =  randomized controlled trials, NRCT =  non-randomized controlled trials, NR =  not reported.

Outcome measures: A =  bleeding-related complications, B =  catheter-related infection,

C =  catheter retention time, D =  catheter occlusion, E = hemodialysis blood flow (mL/min).

### Risk of bias in the literature

The generation of random sequences in three RCTs [13, 22, 23] were regarded as unclear. The allocation of the random scheme was as unclear in two RCTs[13, 23]. Other items were judged as low risk bias in RCTs. Three cohort studies [12,20,21] were considered high-quality articles. For comparability, considering some possible confounding factors between the exposed and non-exposed groups (such as coagulation parameters before intervention, intubation site, history of diabetes, and previous CVC insertion), we give 1 point for two studies. Among the outcomes, we give a score of 0 because neither study had a long follow-up. The total risk score for two articles were 7 points. The overall risk of bias in the NRCT [19] were moderate. In the domain of confounding bias, due to some important possible confounding factors (such as age, gender, coagulation parameters before intervention, intubation site, history of diabetes and previous CVC insertion history), we judged that there was a moderate risk of bias in the study. For the domain of bias due to deviations from intended intervention, we judged it to be at moderate risk of bias. Because of uncertainty about whether patients were receiving other anticoagulant therapy during hemodialysis treatment, we considered that there might be an imbalance across intervention groups for the important co-interventions. Other domains had low risk of bias in the NRCT. Bias summary of included studies were shown in S2 Table.

### Catheter occlusione

**Fig 2:**
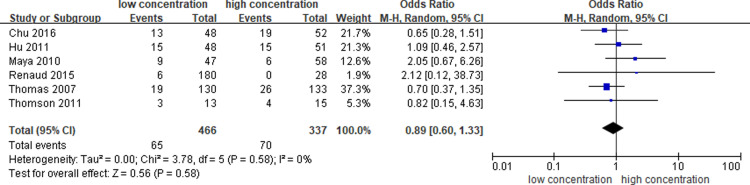
Meta-analysis of Catheter occlusion.

The incidence of catheter occlusion was reported in six studies [12,13,19,21–23] in our meta-analysis, and the pooled OR between different concentrations (1000 U/ml, 5000 U/ml and 10000 U/ml) of heparin-locking solution was 0.89 (95% CI: 0.60–1.33, P = 0.58), with low heterogeneity (I^2^ = 0%). (Fig 2.)

### Hemodialysis blood flow

**Fig 3:**
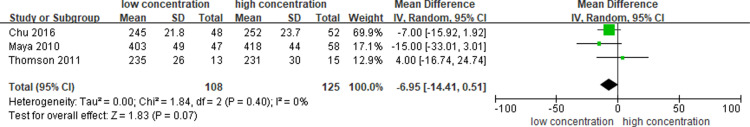
Meta-analysis of hemodialysis blood flow.

Three studies [13,21,22] reported blood flow on hemodialysis, and meta-analysis showed that no significant difference between 1000U/ml and 5000U/ml heparin-locking solution on the blood flow of central venous catheter hemodialysis (MD = -6.95, 95% CI: -14.41– 0.51, P = 0.07), with low heterogeneity (P = 0.40, I^2^ = 0%). (Fig 3.)

### Catheter retention time

**Fig 4:**
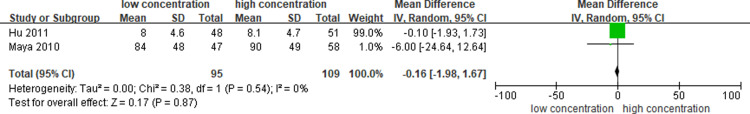
Meta-analysis of catheter retention time.

Catheter retention time was reported in two studies [21,23]. No significant difference in the prevention of catheter retention time between different concentrations of heparin-locking solution (MD = -0.16, 95%CI: -1.98 - 1.67, P = 0.87), and the heterogeneity was low(I^2^ = 0%). (Fig 4.)

### Bleeding-related complications

**Fig 5:**
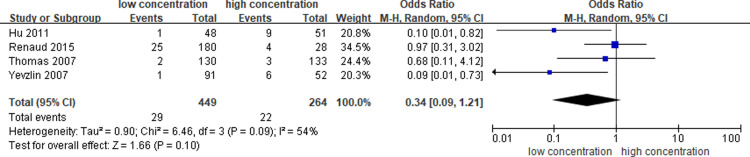
Meta-analysis of bleeding-related complications.

Four articles [12,19,20,23] reported the incidence of bleeding-related complications, and the pooled OR on the bleeding rate between different concentrations (1000 U/ml, 5000 U/ml and 10000 U/ml) of heparin-locking solution was 0.34 (95% CI: 0.09–1.21,), with moderate heterogeneity (I^2^ = 54%). (Fig 5.)

**Fig 6:**
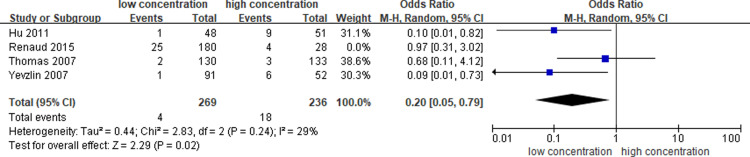
Sensitivity analysis of bleeding-related complications.

In the sensitivity analysis, the heterogeneity was significantly decreased after the removal of one article [12] (I^2^ =  29%). The pooled OR on the bleeding rate between different concentrations (1000 U/ml, 5000 U/ml and 10000 U/ml) of heparin-locking solution was 0.20 (95% CI: 0.05–0.79, P = 0.02) (Fig 6.) This could be attributed to the study by Renaud et al. [12], which included patients at low risk for early complications associated with tunneled dialysis catheters, as the cohort did not consist of those requiring exchanges, reinsertions, or femoral insertions.

### Catheter-related infection

**Fig 7:**
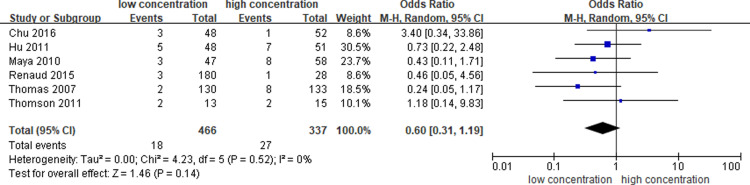
Meta-analysis of catheter-related infection.

Six studies [12,13,19,21–23] reported the incidence of catheter-related infection. Meta-analysis showed that no significant difference in the prevention of catheter-related infection between different concentrations of heparin-locking solution (OR = 0.60, 95%CI: 0.31-1.19, P = 0.14), and the heterogeneity was low (P = 0.52, I^2^ = 0%). (Fig 7.)

## Discussion

Compared with previous meta-analyses, our study included one new RCT study after 2014 and more cohort studies. Our meta-analysis found that 1000 U/ml heparin-locking solution for CVC in hemodialysis patients could decrease the occurrence of bleeding-related complications, without influencing the occurrence of catheter occlusion, hemodialysis blood flow, catheter retention time and catheter-related infection. This is the innovative finding of our meta-analysis. In Han et al’ s meta-analysis [10], it demonstrated that low concentrations ( < 5000 U/ml) of heparin-locking solution could significantly reduce the occurrence of bleeding-related complications, which was consistent with our findings. According to the current evidence [10, 11], we did not detect differences in hemodialysis catheters efficacy between different concentrations of heparin-locking solution.

The increase in heparin-locking solution concentration can lead to elevated systemic activated partial thromboplastin time (APTT), and prolonged APTT is known to lead to an increased risk of bleeding. An RCT study showed [24] that APTT 2 h after catheter placement was prolonged nearly 2 times when high concentration of heparin-locking solution (5000 U/ml) was used. Tan et al ‘s [11] meta-analysis showed that lower concentration (1000U/ml) of heparin-locking solution was superior in shortening APTT, but no significant difference was found in reducing bleeding rates which was different from our findings.

Our study showed that there were no differences in catheter occlusion, hemodialysis blood flow, or catheter retention time among different concentrations of heparin-locking solutions. The longest follow-up period in the studies included was six months,[19] and that maybe a reason for no difference in catheter retention time. For catheter occlusion, 1000U/ml of heparin-locking solution was sufficient to guarantee the patency of the catheter according to our findings. Our study also found no difference among different concentrations of heparin-locking solutions in preventing catheter-related infection. It may be because the follow-up time of the included studies were too short to reveal related adverse events. Given that tunneled CVC can be placed for several months according to the needs of hemodialysis patients, it cannot be ruled out that there could be a high incidence of catheter-related infection during long-term follow-up.

Although three different concentrations of heparin-locking solution were included in our study, statistical heterogeneity in the main outcomes of efficacy (catheter occlusion, hemodialysis blood flow, and catheter retention time) and safety (catheter-related infection) was low (I^2^ = 0%). Only bleeding-related complications showed moderate statistical heterogeneity (I^2^ = 29%), possibly due to the different clinical conditions of the patients included in the four studies.

Several limitations of this study warrant consideration. The imprecision stemming from the diversity in study designs, along with the limited inclusion of only seven studies, diminishes our overall confidence in the pooled effect estimates. Firstly, there is a notable absence of high-quality RCTs among the included studies. The existing RCTs exhibit issues related to non-standard randomization, which may contribute to selection bias between the experimental and control groups prior to the administration of interventions. Furthermore, the potential for confounding bias is heightened due to the lack of control for confounders associated with the outcomes. Specifically, factors such as catheter type, catheter location, and the timing of catheter insertion may influence outcomes such as catheter-related infections or bleeding. Evidence suggests that heparin coating can enhance catheter patency and diminish the necessity for thrombolytic therapy [21]. Hierarchical or multivariate regression analyses could be employed in future research to account for significant confounding variables related to outcomes. Additionally, the follow-up period in the studies included in this analysis was insufficient, with a minimum duration of only 14 days. This short follow-up may hinder the accurate assessment of outcomes, as the formation and progression of thrombus, fibrin sheaths, and other factors leading to catheter occlusion may not be adequately captured within such a brief timeframe. There is a pressing need for high-quality randomized controlled trials and cohort studies with extended follow-up periods in future research endeavors.

## Conclusion

Our findings suggest that a heparin-locking solution at a concentration of 1000 U/ml for central venous catheters (CVC) in hemodialysis patients may reduce the incidence of bleeding-related complications, without impacting the rates of catheter occlusion, hemodialysis blood flow, catheter retention time, or catheter-related infections. However, due to the limited number of included studies, high-quality randomized controlled trials and cohort studies with long-term follow-up are warranted to further validate these results.

## Supporting information

Table S1 The list of keywords used for literature search.
(DOCX)

Table S2 Bias Summary of Included Studies.
(DOCX)

Table S3 The Minimal data set.
(DOCX)

Table S4 Studies identified in the literature search.
(DOCX)

Table S5 PRISMA checklist.
(DOCX)
