# Supplementary material for: Effect of different concentrations of heparin-locking solution for central venous catheters in hemodialysis patients: A systematic review and meta-analysis
Source: PLoS One. 2025 Mar 25;20(3):e0320207. doi: 10.1371/journal.pone.0320207 (PMC11936217; doi:10.1371/journal.pone.0320207)
Supplement: Table S1 — (DOCX) [file pone.0320207.s001.docx]

Effect of different concentrations of heparin-locking solution for central venous catheters in hemodialysis patients: A systematic review and meta-analysis

Supporting Information

List of Keywords for Literature Search

***Search-String Central Venous Catheters***

"Catheter, Central Venous" OR "Catheters, Central Venous" OR "Venous Catheter, Central" OR "Venous Catheters, Central" OR "Central Venous Catheter"

***Search-String renal dialysis***

"Hemodialysis" OR "Dialyses, Renal" OR "Renal Dialyses" OR "Dialysis, Extracorporeal" OR "Dialyses, Extracorporeal" OR "Extracorporeal Dialyses" OR "Extracorporeal Dialysis"

***Search-String Heparin***

"Unfractionated Heparin" OR "Heparin, Unfractionated" OR Liquaemin OR "Sodium Heparin" OR "Heparin, Sodium" OR "Heparin Sodium" OR "alpha-Heparin" OR "alpha Heparin"

We searched the databases PubMed, Embase, Web of science and the Cochrane Library and Clinical Trial Database (clinicaltrials.gov). For each of the databases we ran the above keyword search with separate title, keywords, and abstract filters for each search-string.
