# Supplementary material for: Effect of different concentrations of heparin-locking solution for central venous catheters in hemodialysis patients: A systematic review and meta-analysis
Source: PLoS One. 2025 Mar 25;20(3):e0320207. doi: 10.1371/journal.pone.0320207 (PMC11936217; doi:10.1371/journal.pone.0320207)
Supplement: Table S2 — (DOCX) [file pone.0320207.s002.docx]

Effect of different concentrations of heparin-locking solution for central venous catheters in hemodialysis patients: A systematic review and meta-analysis

Supporting Information

Bias Summary of Included Studies

**Study: CHANDRA M. THOMAS 2007 (NRCT)**

| **Bias domain** | **Signalling questions** | **Elaboration** | **Response options** |
| --- | --- | --- | --- |
| **Bias due to confounding** | | | |
|  | 1.1 Is there potential for confounding of  the effect of intervention in this study?  If N/PN to 1.1: the study can be  considered to be at low risk of bias due to  confounding and no further signalling  questions need be considered  If Y/PY to 1.1: determine whether there is  a need to assess time-varying confounding: | PY | Some important and potentially confounding information was missing: the coagulation status of the patient before the intervention, the insertion site of the catheter. |
|  | 1.2. Was the analysis based on splitting  participants’ follow up time according to  intervention received?  If N/PN, answer questions relating to  baseline confounding (1.4 to 1.6)  If Y/PY, go to question 1.3. | PN | Patients who received the intervention were followed for two separate periods, which were not analyzed |
|  | 1.3. Were intervention discontinuations  or switches likely to be related to factors  that are prognostic for the outcome?  If N/PN, answer questions relating to  baseline confounding (1.4 to 1.6)  If Y/PY, answer questions relating to both  baseline and time-varying confounding  (1.7 and 1.8) | NA |  |
|  | **Questions relating to baseline confounding only** | | |
|  | 1.4. Did the authors use an appropriate  analysis method that controlled for all the  important confounding domains? | PY | A parametric survival model with a Weibull distribution was fitted using gamma shared frailty to account for possible dependence between repeated observations (for subjects in both study periods). Variables considered in the model included heparin study period, age, catheter type (non-tunneled or tunneled), CVC duration, and others. |
|  | 1.5. If Y/PY to 1.4: Were confounding  domains that were controlled for  measured validly and reliably by the  variables available in this study? | PY |  |
|  | 1.6. Did the authors control for any post-  intervention variables that could have  been affected by the intervention? | Y | Patients were excluded if their  catheters were locked with a solution other than heparin or if there was evidence of catheter malfunction (defined as a mean blood flow 250 mL/min during the prior dialysis session) |
|  | **Questions relating to baseline and time-varying confounding** | | |
|  | 1.7. Did the authors use an appropriate  analysis method that controlled for all the  important confounding domains and for  time-varying confounding? | Y | Fit a parametric survival model with Weibull distribution using gamma shared frailty to account for the possible dependence between repeated observations (for subjects who were in both study periods). |
|  | 1.8. If Y/PY to 1.7: Were confounding  domains that were controlled for  measured validly and reliably by the  variables available in this study? | PY |  |
|  | **Risk of bias judgement** | **Moderate** |  |
|  | Optional: What is the predicted direction  of bias due to confounding? |  |  |
| **Bias in selection of participants into the study** | | | |
|  | 2.1. Was selection of participants into the  study (or into the analysis) based on  participant characteristics observed after  the start of intervention?  If N/PN to 2.1: go to 2.4 | PN | Is a retrospective cohort study with baseline and outcome data collected from all patients who met the inclusion criteria |
|  | 2.2. If Y/PY to 2.1: Were the post-  intervention variables that influenced  selection likely to be associated with  intervention? | NA |  |
|  | 2.3 If Y/PY to 2.2: Were the postintervention variables that influenced selection likely to be influenced by the outcome or a cause of the outcome? | NA |  |
|  | 2.4. Do start of follow-up and start of intervention coincide for most participants? | Y | A 6-month observation period for the heparin 10,000 U/ml locking solution  and a 3-month observation period for the heparin 1,000 U/ml locking solution |
|  | 2.5. If Y/PY to 2.2 and 2.3, or N/PN to  2.4: Were adjustment techniques used that are likely to correct for the presence of selection biases? | NA |  |
|  | **Risk of bias judgement** | **Low** |  |
|  | Optional: What is the predicted direction of bias due to selection of participants into the study? |  |  |
| **Bias in classification of interventions** | | | |
|  | 3.1 Were intervention groups clearly defined? | Y | Two separate time periods were studied: a 6-month observation period for the heparin 10,000 U/ml locking solution (April 1, 2003, to September 30, 2003) and a 3-month observation period for the heparin 1,000 U/ml locking solution (November 1, 2003, to January 31, 2004). |
|  | 3.2 Was the information used to define intervention groups recorded at the start of the intervention? | Y | Clinical and demographic data were collected through review of the hemodialysis chart and the SARP database. Details regarding catheter function at each hemodialysis session  were abstracted from the patient’s hemodialysis chart. |
|  | 3.3 Could classification of intervention status have been affected by knowledge of the outcome or risk of the outcome? | N | Two separate time periods were studied: a 6-month observation period for the heparin 10,000 U/ml locking solution (April 1, 2003, to September 30, 2003) and a 3-month observation period for the heparin 1,000 U/ml locking solution (November 1, 2003, to January 31, 2004). |
|  | **Risk of bias judgement** | **Low** |  |
|  | Optional: What is the predicted direction of bias due to measurement of outcomes or interventions? |  |  |
| **Bias due to deviations from intended interventions** | | | |
|  | If your aim for this study is to assess the effect of assignment to intervention, answer questions 4.1 and 4.2 | |  |
|  | 4.1. Were there deviations from the intended intervention beyond what would be expected in usual practice? | NA |  |
|  | 4.2. If Y/PY to 4.1: Were these deviations from intended intervention unbalanced between groups and likely to have affected the outcome? | NA |  |
|  | If your aim for this study is to assess the effect of starting and adhering to intervention, answer questions 4.3 to 4.6 | |  |
|  | 4.3. Were important co-interventions balanced across intervention groups? | PN | No co-interventions. |
|  | 4.4. Was the intervention implemented successfully for most participants? | Y | A retrospective study |
|  | 4.5. Did study participants adhere to the assigned intervention regimen? | Y | A retrospective study |
|  | 4.6. If N/PN to 4.3, 4.4 or 4.5: Was an appropriate analysis used to estimate the effect of starting and adhering to the intervention? | NA |  |
|  | **Risk of bias judgement** | **Moderate** |  |
|  | Optional: What is the predicted direction of bias due to deviations from the intended interventions? |  |  |
| **Bias due to missing data** | | | |
|  | 5.1 Were outcome data available for all, or nearly all, participants? | Y | A retrospective study. Clinical and demographic data were collected through review of the hemodialysis chart and the SARP database. |
|  | 5.2 Were participants excluded due to missing data on intervention status? | PN |  |
|  | 5.3 Were participants excluded due to missing data on other variables needed for the analysis? | PN |  |
|  | 5.4 If PN/N to 5.1, or Y/PY to 5.2 or 5.3:  Are the proportion of participants and reasons for missing data similar across interventions? | NA |  |
|  | 5.5 If PN/N to 5.1, or Y/PY to 5.2 or 5.3: Is  there evidence that results were robust to  the presence of missing data? | NI |  |
|  | **Risk of bias judgement** | **Low** |  |
|  | Optional: What is the predicted direction  of bias due to missing data? |  |  |
| **Bias in measurement of outcomes** | | | |
|  | 6.1 Could the outcome measure have been influenced by knowledge of the intervention received? | PY | In conclusion, the use of a  lower concentration of heparin was not associated with an  increased risk of catheter malfunction but may be associated  with greater rt-PA use. |
|  | 6.2 Were outcome assessors aware of the intervention received by study participants? | NI | Information on outcome assessors was not provided, but the primary outcome of catheter failure (based on dialysis blood flow) and the secondary outcome of bleeding-related complications were less likely to be influenced by outcome assessors |
|  | 6.3 Were the methods of outcome assessment comparable across intervention groups? | Y | The primary outcome was a composite outcome of catheter malfunction defined as a mean blood flow 250 mL/min during two consecutive dialysis sessions. The secondary out  come was the rate of admission to hospital with a bleeding  related complication. |
|  | 6.4 Were any systematic errors in measurement of the outcome related to intervention received? | N |  |
|  | **Risk of bias judgement** | **Low** |  |
|  | Optional: What is the predicted direction  of bias due to measurement of  outcomes? |  |  |
| **Bias in selection of the reported result** | | | |
|  | Is the reported effect estimate likely to be  selected, on the basis of the results, from...  7.1. ... multiple outcome measurements within the outcome domain? |  |  |
|  |  | N | Outcome measures are unlikely to be measured in multiple ways |
|  | 7.2 ... multiple analyses of the  intervention-outcome relationship? | Y | The crude rate of catheter failure was calculated, and the analysis was repeated in order to determine whether correlations between subjects in the two study periods could affect the results |
|  | 7.3 ... different subgroups? | N |  |
|  | **Risk of bias judgement** | **Low** |  |
|  | Optional: What is the predicted direction  of bias due to selection of the reported  result? |  |  |
| **Overall bias** | | | |
|  | **Risk of bias judgement** | **Moderate** |  |
|  | Optional: What is the overall predicted direction of bias for this outcome? |  |  |

**Three Cohort studies.**

| **Studies** | **Selection** | | | | **Comparability** | | **Outcome** | | | **Quality grade** |
| --- | --- | --- | --- | --- | --- | --- | --- | --- | --- | --- |
|  | Representativeness of the Exposed Cohort | Selection of the Non-  Exposed Cohort | Ascertainment of Exposure | Demonstration That Outcome of Interest Was Not Present at Start of Study | Comparability of cohorts on the basis of the design or analysis | Comparability of cohorts on the basis of the measurement | Outcomes were assessed accurately and without bias | Was Follow-Up Long Enough for Outcomes to Occur | Adequacy of Follow Up of Cohorts |  |
| **Yevzlin et al.** | 1 | 1 | 1 | 1 | 0.5 | 0.5 | 1 | 0 | 1 | **High** |
|  | The sample can represen-tative of the popula-  tion | The baseline conditions were consistent and all were free of disease | Exposure factors were recorded objectively | Exposure factors were recorded objectively | Exposure factors were recorded objectively | Differences in some confounding factors | Results were obtained from the hospital medical record system | Follow-up periods are not reported | All participants completed the follow-up |  |
| **Maya et al.** | 1 | 1 | 1 | 1 | 0.5 | 0.5 | 1 | 0 | 1 | **High** |
|  | The sample can represen-tative of the popula-  tion | The baseline conditions were consistent and all were free of disease | Exposure factors were recorded objectively | Exposure factors were recorded objectively | Exposure factors were recorded objectively | Differences in some confounding factors | Results were obtained from the hospital medical record system | 3 months follow-up period | All participants completed the follow-up |  |
| **Maya et al.** | 1 | 1 | 1 | 1 | 0.5 | 0.5 | 1 | 0 | 1 | **High** |
|  | The sample can represen-tative of the popula-  tion | The baseline conditions were consistent and all were free of disease | Exposure factors were recorded objectively | Exposure factors were recorded objectively | Exposure factors were recorded objectively | Differences in some confounding factors | Results were obtained from the hospital medical record system | 30 days follow-up period | All participants completed the follow-up |  |

**Three RCTs**

| **Studies** | **Randomization** | **Allocation bias** | **Performance bias** | **Detection bias** | **Attrition bias** | **Reporting**  **bias** | **Other bias** |
| --- | --- | --- | --- | --- | --- | --- | --- |
| **Hu et al.** | **Unclear risk of**  **bias** | **Unclear risk of**  **bias** | **Low risk of bias** | **Low risk of bias** | **Low risk of bias** | **Low risk of bias** | **Low risk of bias** |
|  | Insufficient  information about the sequence generation  process to permit  judgement of ‘Low risk’ or ‘High risk’ | “...heparin solution was randomly chosen for retention.” | Outcomes were not affected by the lack of blinding | Outcomes were not affected by the lack of blinding | No missing  outcome data  reported | All outcomes  specified in the  methods were  reported | No evidence of  other bias. |
| **Thomson et al.** | **Unclear risk of**  **bias**  **]** | **Low risk of bias** | **Low risk of bias** | **Low risk of bias** | **Low risk of bias** | **Low risk of bias** | **Low risk of bias** |
|  | “patients were randomly allocated...”Insufficient  information about the sequence generation  process to permit  judgement of ‘Low risk’ or ‘High risk’ | “All members of the investigating team were blinded to the allocation | Outcomes were not affected by the lack of blinding | Outcomes were not affected by the lack of blinding | No missing  outcome data  reported | All outcomes  specified in the  methods were  reported | No evidence of  other bias. |
| **Chu et al.** | **Unclear risk of**  **bias** | **Unclear risk of**  **bias** | **Low risk of bias** | **Low risk of bias** | **Low risk of bias** | **Low risk of bias** | **Low risk of bias** |
|  | No relevant quote found in  article | No relevant quote found in  article | Outcomes were not affected by the lack of blinding | Outcomes were not affected by the lack of blinding | No missing  outcome data  reported | All outcomes  specified in the  methods were  reported | No evidence of  other bias. |
