# Supplementary material for: Effect of different concentrations of heparin-locking solution for central venous catheters in hemodialysis patients: A systematic review and meta-analysis
Source: PLoS One. 2025 Mar 25;20(3):e0320207. doi: 10.1371/journal.pone.0320207 (PMC11936217; doi:10.1371/journal.pone.0320207)
Supplement: Table S3 — (DOCX) [file pone.0320207.s003.docx]

Effect of different concentrations of heparin-locking solution for central venous catheters in hemodialysis patients: A systematic review and meta-analysis

Supporting Information

Minimal data set

***Study Title: Concentration of Heparin-Locking Solution and Risk of***

***Central Venous Hemodialysis Catheter Malfunction***

Characteristics of Research Subjects

| First author | CHANDRA M. THOMAS |
| --- | --- |
| Year of publication | 2007 |
| Publication type | Journal |
| Country | Canada |
| Study period heparin concentrations | Low concentration: 1000U/ml  High concentration: 10000U/ml |
| Number of patients  (Low concentration/High concentration) | 263 (130/133) |
| Age  (Mean ± SD) | Low concentration: 64 ± 17  High concentration: 65 ± 14 |
| Gender  (Male/Female) | Low concentration: 69/61  High concentration: 65/68 |
| Catheter type  (tunneled/non-tunneled) | Low concentration: 98/32  High concentration: 131/32 |
| Follow-up period | A 6-month observation period for the heparin 10,000 U/ml locking solution  and a 3-month observation period for the heparin 1,000 U/ml locking solution |
| Catheter occlusion  (Events/Total) D | Low concentration: 19/130  High concentration: 26/133 |
| Bleeding-related complications  (Events/Total) A | Low concentration: 2/130  High concentration: 3/133 |
| Catheter-related infection  (Events/Total) B | Low concentration: 2/130  High concentration: 8/133 |
| Catheter retention time  (Events/Total) C | NR |
| Hemodialysis blood flow (mL/min)  (Mean ± SD) E | NR |

***Study Title: Concentrated Heparin Lock Is Associated with Major Bleeding Complications after Tunneled Hemodialysis Catheter Placement***

Characteristics of Research Subjects

| First author | Alexander S. Yevzlin |
| --- | --- |
| Year of publication | 2007 |
| Publication type | Journal |
| Country | USA |
| Study period heparin concentrations | Low concentration: 1000U/ml  High concentration: 5000U/ml |
| Number of patients  (Low concentration/High concentration) | 143 (91/52) |
| Age  (Mean ± SD) | Low concentration: 55.99 ± 16.0  High concentration: 56.15 ± 16.7 |
| Gender  (Male/Female) | Low concentration: 61/30  High concentration: 29/23 |
| Catheter type  (tunneled/non-tunneled) | Low concentration: 91/0  High concentration: 52/0 |
| Follow-up period | NR |
| Catheter occlusion  (Events/Total) D | NR |
| Bleeding-related complications  (Events/Total) A | Low concentration: 1/91  High concentration: 6/52 |
| Catheter-related infection  (Events/Total) B | NR |
| Catheter retention time  (Events/Total) C | NR |
| Hemodialysis blood flow (mL/min)  (Mean ± SD) E | NR |

***Study Title: Does the Heparin Lock Concentration Affect Hemodialysis***

***Catheter Patency?***

Characteristics of Research Subjects

| First author | Ivan D. Maya |
| --- | --- |
| Year of publication | 2010 |
| Publication type | Journal |
| Country | USA |
| Study period heparin concentrations | Low concentration: 1000U/ml  High concentration: 5000U/ml |
| Number of patients  (Low concentration/High concentration) | 105 (47/58) |
| Age  (Mean ± SD) | Low concentration: 55 ± 14  High concentration: 53± 15 |
| Gender  (Male/Female) | Low concentration: 27/20  High concentration: 37/21 |
| Catheter type  (tunneled/non-tunneled) | Low concentration: 47/0  High concentration: 58/0 |
| Follow-up period | 3 months |
| Catheter occlusion  (Events/Total) D | Low concentration: 9/47  High concentration: 6/58 |
| Bleeding-related complications  (Events/Total) A | NR |
| Catheter-related infection  (Events/Total) B | Low concentration: 3/47  High concentration: 8/58 |
| Catheter retention time  (Mean ± SD) C | Low concentration: 84 ± 48  High concentration: 90± 49 |
| Hemodialysis blood flow (mL/min)  (Mean ± SD) E | Low concentration: 403 ± 49  High concentration: 418 ± 44 |

***Study Title: Low-Dose Heparin Retention in Temporary Hemodialysis Double-Lumen Catheter Does Not Increase Catheter Occlusion and Might Reduce***

***Risk of Bleeding***

Characteristics of Research Subjects

| First author | HaoHuan Hu |
| --- | --- |
| Year of publication | 2010 |
| Publication type | Journal |
| Country | China |
| Study period heparin concentrations | Low concentration: 1000U/ml  High concentration: 5000U/ml |
| Number of patients  (Low concentration/High concentration) | 99 (48/51) |
| Age  (Mean ± SD) | Low concentration: 67 ± 15  High concentration: 68 ± 14 |
| Gender  (Male/Female) | Low concentration: 27/21  High concentration: 40/11 |
| Catheter type  (tunneled/non-tunneled) | Low concentration: 0/48  High concentration: 0/51 |
| Follow-up period | 14 days or until catheter |
| Catheter occlusion  (Events/Total) D | Low concentration: 15/48  High concentration: 15/51 |
| Bleeding-related complications  (Events/Total) A | Low concentration: 1/48  High concentration: 9/51 |
| Catheter-related infection  (Events/Total) B | Low concentration: 5/48  High concentration: 7/51 |
| Catheter retention time  (Mean ± SD) C | Low concentration: 8.0 ± 4.6  High concentration: 8.1 ± 4.7 |
| Hemodialysis blood flow (mL/min)  (Mean ± SD) E | NR |

***Study Title: The effect of heparinized catheter lock solutions on systemic anticoagulation in hemodialysis patients***

Characteristics of Research Subjects

| First author | P.C. Thomson |
| --- | --- |
| Year of publication | 2011 |
| Publication type | Journal |
| Country | UK |
| Study period heparin concentrations | Low concentration: 1000U/ml  High concentration: 5000U/ml |
| Number of patients  (Low concentration/High concentration) | 28 (13/15) |
| Median age | 68 years (range 40.2–84.8) |
| Gender  (Male/Female) | Low concentration: 7/6  High concentration: 9/6 |
| Catheter type  (tunneled/non-tunneled) | Low concentration: 0/13  High concentration: 0/15 |
| Follow-up period | Until catheter removal (7 days) |
| Catheter occlusion  (Events/Total) D | Low concentration: 3/13  High concentration: 4/15 |
| Bleeding-related complications  (Events/Total) A | NR |
| Catheter-related infection  (Events/Total) B | Low concentration: 2/13  High concentration: 2/15 |
| Catheter retention time  (Mean ± SD) C | NR |
| Hemodialysis blood flow (mL/min)  (Mean ± SD) E | Low concentration: 235 ± 26  High concentration: 231 ± 30 |

***Study Title: Effect of ultra-low dose and standard heparin locks on early tunnelled dialysis catheter outcomes in low-risk dialysis patients***

Characteristics of Research Subjects

| First author | CLAUDE J RENAUD |
| --- | --- |
| Year of publication | 2015 |
| Publication type | Journal |
| Country | Singapore |
| Study period heparin concentrations | Low concentration: 1000U/ml  High concentration: 5000U/ml |
| Number of patients  (Low concentration/High concentration) | 208 (180/28) |
| Age  (Mean ± SD) | Low concentration: 59 ± 12  High concentration: 61 ± 11 |
| Gender  (Male/Female) | Low concentration: 77 / 73  High concentration: 21 / 7 |
| Catheter type  (tunneled/non-tunneled) | Low concentration: 180 / 0  High concentration: 28 / 0 |
| Follow-up period | 30days |
| Catheter occlusion  (Events/Total) D | Low concentration: 6/180  High concentration: 0/28 |
| Bleeding-related complications  (Events/Total) A | Low concentration: 25/180  High concentration: 4/28 |
| Catheter-related infection  (Events/Total) B | Low concentration: 3/180  High concentration: 1/28 |
| Catheter retention time  (Mean ± SD) C | NR |
| Hemodialysis blood flow (mL/min)  (Mean ± SD) E | NR |

***Study Title: Low dose heparin lock (1000 U/mL) maintains tunnelled hemodialysis catheter patency when compared with high dose heparin (5000 U/mL): A randomised controlled trial***

Characteristics of Research Subjects

| First author | Ginger CHU |
| --- | --- |
| Year of publication | 2016 |
| Publication type | Journal |
| Country | Australia |
| Study period heparin concentrations | Low concentration: 1000U/ml  High concentration: 5000U/ml |
| Number of patients  (Low concentration/High concentration) | 100 (48/52) |
| Age  (Mean ± SD) | Low concentration: 62 ± 18.3  High concentration: 62 ± 17.0 |
| Gender  (Male/Female) | NR |
| Catheter type  (tunneled/non-tunneled) | Low concentration: 48/0  High concentration: 52/0 |
| Follow-up period | 90days |
| Catheter occlusion  (Events/Total) D | Low concentration: 13/48  High concentration: 19/52 |
| Bleeding-related complications  (Events/Total) A | NR |
| Catheter-related infection  (Events/Total) B | Low concentration: 3/48  High concentration: 1/52 |
| Catheter retention time  (Mean ± SD) C | NR |
| Hemodialysis blood flow (mL/min)  (Mean ± SD) E | Low concentration: 245 ± 21.8  High concentration: 252 ± 23.7 |
