# Supplementary material for: Effect of different concentrations of heparin-locking solution for central venous catheters in hemodialysis patients: A systematic review and meta-analysis
Source: PLoS One. 2025 Mar 25;20(3):e0320207. doi: 10.1371/journal.pone.0320207 (PMC11936217; doi:10.1371/journal.pone.0320207)
Supplement: Table S4 — (DOCX) [file pone.0320207.s004.docx]

Effect of different concentrations of heparin-locking solution for central venous catheters in hemodialysis patients: A systematic review and meta-analysis

Supporting Information

Studies identified in the literature search

| Number | Title | Exclusion reason/ Included |
| --- | --- | --- |
| 1 | Concentration of Heparin-Locking Solution and Risk of Central Venous Hemodialysis Catheter Malfunction. | Included |
| 2 | The effect of heparinized catheter lock solutions on systemic anticoagulation in hemodialysis patients. | Included |
| 3 | Concentrated Heparin Lock Is Associated with Major Bleeding Complications after Tunneled Hemodialysis Catheter Placement. | Included |
| 4 | Does the Heparin Lock Concentration Affect Hemodialysis Catheter Patency? | Included |
| 5 | Low-Dose Heparin Retention in Temporary Hemodialysis Double-Lumen Catheter Does Not Increase Catheter Occlusion and Might Reduce Risk of Bleeding. | Included |
| 6 | Low concentration of heparin used for permanent catheters canal locking is effective and diminishes the risk of bleeding. | Included |
| 7 | [Low dose heparin lock (1000 U/mL) maintains tunnelled hemodialysis catheter patency when compared with high dose heparin (5000 U/mL): a randomised controlled trial](https://www.cochranelibrary.com/central/doi/10.1002/central/CN-01402215/full). | Included |
| 8 | [Cathasept line lock and microbial colonization of tunneled hemodialysis catheters: a multicenter randomized controlled trial](https://www.cochranelibrary.com/central/doi/10.1002/central/CN-01125999/full). | Irrelevant to the purpose |
| 9 | [Cathasept Line Lock and Microbial Colonization of Tunneled Hemodialysis Catheters: a Multicenter Randomized Controlled Trial](https://www.cochranelibrary.com/central/doi/10.1002/central/CN-01259092/full). | Irrelevant to the purpose |
| 10 | [Concentrated sodium chloride catheter lock solution--a new effective alternative method for hemodialysis patients with high bleeding risk](https://www.cochranelibrary.com/central/doi/10.1002/central/CN-00910990/full). | Irrelevant to the purpose |
| 11 | [An ethanol/sodium citrate locking solution compared to heparin to prevent hemodialysis catheter-related infections: a randomized pilot study](https://www.cochranelibrary.com/central/doi/10.1002/central/CN-01132855/full). | Irrelevant to the purpose |
| 12 | [An ethanol/sodium citrate locking solution compared to heparin to prevent hemodialysis catheter-related infections: a randomized pilot study](https://www.cochranelibrary.com/central/doi/10.1002/central/CN-01260200/full). | Irrelevant to the purpose |
| 13 | [Clinical application of 4% sodium citrate and heparin in the locking of central venous catheters (excluding dialysis catheters) in intensive care unit patients: a pragmatic randomized controlled trial](https://www.cochranelibrary.com/central/doi/10.1002/central/CN-02578682/full). | Irrelevant to the purpose |
| 14 | [Taurolidine and Heparin as Catheter Lock Solution for Central Venous Catheters in Hemodialysis](https://www.cochranelibrary.com/central/doi/10.1002/central/CN-02702591/full). | Irrelevant to the purpose |
| 15 | [Evaluating the safety and efficacy of argatroban locking solution in the prevention of the dysfunction of haemodialysis central venous catheters: a study protocol for a randomized controlled trial](https://www.cochranelibrary.com/central/doi/10.1002/central/CN-02258017/full). | Irrelevant to the purpose |
| 16 | [Comparison of heparin to citrate as a catheter locking solution for non-tunneled central venous hemodialysis catheters in patients requiring renal replacement therapy for acute renal failure (VERROU-REA study): study protocol for a randomized controlled trial](https://www.cochranelibrary.com/central/doi/10.1002/central/CN-01082862/full). | Irrelevant to the purpose |
| 17 | [Comparison of heparin to citrate as a catheter locking solution for non-tunneled central venous hemodialysis catheters in patients requiring renal replacement therapy for acute renal failure (VERROU-REA study): study protocol for a randomized controlled trial](https://www.cochranelibrary.com/central/doi/10.1002/central/CN-01112368/full). | Irrelevant to the purpose |
| 18 | [Ethanol combined with heparin as a locking solution for the prevention of catheter related blood stream infections in hemodialysis patients: a prospective randomized study](https://www.cochranelibrary.com/central/doi/10.1002/central/CN-01459182/full). | Irrelevant to the purpose |
| 19 | [Effect of taurolidine citrate and unfractionated heparin on inflammatory state and dialysis adequacy in hemodialysis patients](https://www.cochranelibrary.com/central/doi/10.1002/central/CN-02287508/full). | Irrelevant to the purpose |
| 20 | [The CLOCK trial, a double-blinded randomized controlled trial: trisodium citrate 30% and minocycline 3 mg/mL plus EDTA 30 mg/mL are effective and safe for catheter patency maintenance among CKD 5D patients on hemodialysis](https://www.cochranelibrary.com/central/doi/10.1002/central/CN-01401705/full). | Irrelevant to the purpose |
| 21 | [The CLOCK trial, a double-blinded randomized controlled trial: trisodium citrate 30% and minocycline 3 mg/mL plus EDTA 30 mg/mL are effective and safe for catheter patency maintenance among CKD 5D patients on hemodialysis](https://www.cochranelibrary.com/central/doi/10.1002/central/CN-01247311/full). | Irrelevant to the purpose |
| 22 | [Taurolidine/Heparin Lock Solution and Catheter-Related Bloodstream Infection in Hemodialysis: a Randomized, Double-Blind, Active-Control, Phase 3 Study](https://www.cochranelibrary.com/central/doi/10.1002/central/CN-02615778/full). | Irrelevant to the purpose |
| 23 | [Cefazolin-gentamicin versus taurolidine-citrate for the prevention of infection in tunneled central catheters in hemodialysis patients: a quasi-experimental trial](https://www.cochranelibrary.com/central/doi/10.1002/central/CN-01993811/full). | Irrelevant to the purpose |
| 24 | [Malfunctioning temporary hemodialysis catheters in patients with novel coronavirus disease 2019](https://www.cochranelibrary.com/central/doi/10.1002/central/CN-02210544/full). | Irrelevant to the purpose |
| 25 | [A randomized trial comparing gentamicin/citrate and heparin locks for central venous catheters in maintenance hemodialysis patients](https://www.cochranelibrary.com/central/doi/10.1002/central/CN-00804834/full). | Irrelevant to the purpose |
| 26 | [Concentrated citrate locking in order to reduce the long-term complications of central venous catheters: a randomized controlled trial in patients with hematological malignancies](https://www.cochranelibrary.com/central/doi/10.1002/central/CN-01040837/full). | Irrelevant to the purpose |
| 27 | [Concentrated citrate locking in order to reduce the long-term complications of central venous catheters: a randomized controlled trial in patients with hematological malignancies](https://www.cochranelibrary.com/central/doi/10.1002/central/CN-01115749/full). | Irrelevant to the purpose |
| 28 | [Sodium citrate versus heparin catheter locks for cuffed central venous catheters: a single-center randomized controlled trial](https://www.cochranelibrary.com/central/doi/10.1002/central/CN-00698178/full). | Irrelevant to the purpose |
| 29 | [Taurolidine-based catheter lock regimen significantly reduces overall costs, infection, and dysfunction rates of tunneled hemodialysis catheters](https://www.cochranelibrary.com/central/doi/10.1002/central/CN-01665707/full). | Irrelevant to the purpose |
| 30 | [Natural saline-flush is sufficient to maintain patency of immobilized-urokinase double-lumen catheter used to provide temporary blood access for hemodialysis](https://www.cochranelibrary.com/central/doi/10.1002/central/CN-00504277/full). | Irrelevant to the purpose |
| 31 | [Cov-hep study: heparin in standard anticoagulation based on citrate for continuous veno-venous hemodialysis in patients with COVID-19: a structured summary of a study protocol for a randomized controlled trial](https://www.cochranelibrary.com/central/doi/10.1002/central/CN-02210848/full). | Irrelevant to the purpose |
| 32 | [Comparison of alteplase and heparin in maintaining the patency of paediatric central venous haemodialysis lines: a randomised controlled trial](https://www.cochranelibrary.com/central/doi/10.1002/central/CN-00586091/full). | Irrelevant to the purpose |
| 33 | [A randomized controlled trial of gentamicin/citrate versus heparin locks for central venous catheters in maintenance hemodialysis](https://www.cochranelibrary.com/central/doi/10.1002/central/CN-01912574/full). | Irrelevant to the purpose |
| 34 | Improvement of a chronic rat model for peritoneal dialysis by using heparin-coated catheters. | Irrelevant to the purpose |
| 35 | [Bicarbonate vs Heparin Catheter Lock in Chronic Hemodialysis Patients](https://www.cochranelibrary.com/central/doi/10.1002/central/CN-01966379/full). | Irrelevant to the purpose |
| 36 | [Minocycline-EDTA lock solution prevents catheter-related bacteremia in hemodialysis](https://www.cochranelibrary.com/central/doi/10.1002/central/CN-00804490/full). | Irrelevant to the purpose |
| 37 | [A comparative prospective study on the use of low concentrate citrate lock versus heparin lock in permanent dialysis catheters](https://www.cochranelibrary.com/central/doi/10.1002/central/CN-00387718/full). | Irrelevant to the purpose |
| 38 | [Filling hemodialysis catheters in the interdialytic period: heparin versus citrate versus polygeline: a prospective randomized study](https://www.cochranelibrary.com/central/doi/10.1002/central/CN-00157063/full). | Irrelevant to the purpose |
| 39 | [Approaches to prolong the use of uncuffed hemodialysis catheters: results of a randomized trial](https://www.cochranelibrary.com/central/doi/10.1002/central/CN-00786246/full). | Irrelevant to the purpose |
| 40 | [Efficacy and safety of enoxaparin during hemodialysis: results from the HENOX study](https://www.cochranelibrary.com/central/doi/10.1002/central/CN-00891990/full). | Irrelevant to the purpose |
| 41 | [A randomised controlled trial of Heparin versus EthAnol Lock THerapY for the prevention of Catheter Associated infecTion in Haemodialysis patients--the HEALTHY-CATH trial](https://www.cochranelibrary.com/central/doi/10.1002/central/CN-00878361/full). | Irrelevant to the purpose |
| 42 | [A randomized, controlled trial of a new vascular catheter flush solution (minocycline-EDTA) in temporary hemodialysis access](https://www.cochranelibrary.com/central/doi/10.1002/central/CN-00528926/full). | Irrelevant to the purpose |
| 43 | [Prevention of catheter lumen occlusion with rT-PA versus heparin (Pre-CLOT): study protocol of a randomized trial](https://www.cochranelibrary.com/central/doi/10.1002/central/CN-00564110/full). | Irrelevant to the purpose |
| 44 | [A randomized controlled trial of topical exit site mupirocin application in patients with tunnelled, cuffed haemodialysis catheters](https://www.cochranelibrary.com/central/doi/10.1002/central/CN-00406899/full). | Irrelevant to the purpose |
| 45 | [Prevention of dialysis catheter-related sepsis with a citrate-taurolidine-containing lock solution](https://www.cochranelibrary.com/central/doi/10.1002/central/CN-00460389/full). | Irrelevant to the purpose |
| 46 | [Ultra-low dose heparin locks perform well on non-tunnelled temporary haemodialysis catheters](https://www.cochranelibrary.com/central/doi/10.1002/central/CN-01068671/full). | Irrelevant to the purpose |
| 47 | [Is heparin the best solution? Other options for “locking” catheters](https://www.cochranelibrary.com/central/doi/10.1002/central/CN-01752802/full). | Irrelevant to the purpose |
| 48 | [Efficacy of 5% Trisodium Citrate and Low Dose Heparin as Catheter-Locking Solution for Central Venous Hemodialysis Catheters: a Randomized Controlled Study](https://www.cochranelibrary.com/central/doi/10.1002/central/CN-02626519/full). | Irrelevant to the purpose |
| 49 | [Locking Hemodialysis Catheters With Trimethoprim-Ethanol-Ca-EDTA to Prevent Bloodstream Infections: a Randomized, Evaluator-blinded Clinical Trial](https://www.cochranelibrary.com/central/doi/10.1002/central/CN-01959768/full). | Irrelevant to the purpose |
| 50 | [Patency of arteriovenous fistula for dialysis improbe with topical spraygel heparin](https://www.cochranelibrary.com/central/doi/10.1002/central/CN-00772437/full). | Irrelevant to the purpose |
| 51 | [A randomized controlled trial of comparative effectiveness between sodium bicarbonate and heparin as a locking solution for tunnelled central venous catheters among haemodialysis patients](https://www.cochranelibrary.com/central/doi/10.1002/central/CN-02324321/full). | Irrelevant to the purpose |
| 52 | [A Randomized Controlled Trial of Comparative Efficacy between Sodium Bicarbonate and Heparin as A Locking Solution for Tunneled Central Venous Catheters Among Patients Requiring Maintenance Hemodialysis](https://www.cochranelibrary.com/central/doi/10.1002/central/CN-02338212/full). | Irrelevant to the purpose |
| 53 | [Comparative effectiveness of 30 % trisodium citrate and heparin lock solution in preventing infection and dysfunction of hemodialysis catheters: a randomized controlled trial (CITRIM trial)](https://www.cochranelibrary.com/central/doi/10.1002/central/CN-01365300/full). | Irrelevant to the purpose |
| 54 | [Sodium Bicarbonate versus Heparin as Catheter Lock Solution to Reduce Hemodialysis Catheter Loss due to Catheter Related Thrombosis and Blood Stream Infection](https://www.cochranelibrary.com/central/doi/10.1002/central/CN-02762026/full). | Irrelevant to the purpose |
| 55 | [Comparative effectiveness of 30 % trisodium citrate and heparin lock solution in preventing infection and dysfunction of hemodialysis catheters: a randomized controlled trial (CITRIM trial)](https://www.cochranelibrary.com/central/doi/10.1002/central/CN-01342390/full). | Irrelevant to the purpose |
| 56 | [Prevention of dialysis catheter malfunction with recombinant tissue plasminogen activator](https://www.cochranelibrary.com/central/doi/10.1002/central/CN-00771023/full). | Irrelevant to the purpose |
| 57 | [The effect of heparinized catheter lock solutions on systemic anticoagulation in hemodialysis patients](https://www.cochranelibrary.com/central/doi/10.1002/central/CN-00778919/full). | Irrelevant to the purpose |
| 58 | [Locking tunneled hemodialysis catheters with hypertonic saline (26% NaCl) and heparin to prevent catheter-related bloodstream infections and thrombosis: a randomized, prospective trial](https://www.cochranelibrary.com/central/doi/10.1002/central/CN-00866881/full). | Irrelevant to the purpose |
| 59 | [ENOXAPARIN VERSUS BEMIPARIN IN PERMENANT HEMODIALYSIS CATHETER THROMBOSIS](https://www.cochranelibrary.com/central/doi/10.1002/central/CN-02666824/full). | Irrelevant to the purpose |
| 60 | [Is citrate 4% a safer alternative to heparin in maintaining catheter patency for children vulnerable to systemic bleeding?](https://www.cochranelibrary.com/central/doi/10.1002/central/CN-01421049/full) | Irrelevant to the purpose |
| 61 | [Clinical study on the safety and efficacy of different vascular pathways in patients undergoing continuous renal replacement therapy for maintenance hemodialysis of arteriovenous fistula](https://www.cochranelibrary.com/central/doi/10.1002/central/CN-02555918/full). | Irrelevant to the purpose |
| 62 | [Prophylactic Antimicrobial Catheter Lock](https://www.cochranelibrary.com/central/doi/10.1002/central/CN-01516044/full). | Irrelevant to the purpose |
| 63 | [Comparison of tissue plasminogen activator-antibiotic locks with heparin-antibiotic locks in children with catheter-related bacteraemia](https://www.cochranelibrary.com/central/doi/10.1002/central/CN-00666057/full). | Irrelevant to the purpose |
| 64 | [The impact of catheter-restricted filling with cefotaxime and heparin on the lifespan of temporary hemodialysis catheters: a case controlled study](https://www.cochranelibrary.com/central/doi/10.1002/central/CN-00532490/full). | Irrelevant to the purpose |
| 65 | [A study between two medicated locking solutions in Catheter related infections in patients undergoing dialysis via permacath](https://www.cochranelibrary.com/central/doi/10.1002/central/CN-02498580/full). | Irrelevant to the purpose |
| 66 | [Locking of tunneled hemodialysis catheters with gentamicin and heparin](https://www.cochranelibrary.com/central/doi/10.1002/central/CN-00490240/full). | Irrelevant to the purpose |
| 67 | [Concentrated Citrate Locking to Reduce the Incidence of CVC-related Complications in Hematological Patients](https://www.cochranelibrary.com/central/doi/10.1002/central/CN-01541311/full). | Irrelevant to the purpose |
| 68 | [Safety and efficacy of taurolidine/urokinase versus taurolidine/heparin as a tunneled catheter lock solution in hemodialysis patients: a prospective, randomized, controlled study](https://www.cochranelibrary.com/central/doi/10.1002/central/CN-01982838/full). | Irrelevant to the purpose |
| 69 | [Recombinant tissue plasminogen activator is a useful alternative to heparin in priming quinton permcath](https://www.cochranelibrary.com/central/doi/10.1002/central/CN-00265627/full). | Irrelevant to the purpose |
| 70 | [Dalteparin sodium](https://www.cochranelibrary.com/central/doi/10.1002/central/CN-01776161/full). | Irrelevant to the purpose |
| 71 | [Urokinase: new indication. Thrombosed venous and dialysis catheters: when heparin fails. Urokinase clears about 75% of thrombosed venous catheters intended for long-term use with a dose-related increase in the bleeding risk](https://www.cochranelibrary.com/central/doi/10.1002/central/CN-01720025/full). | Irrelevant to the purpose |
| 72 | [Efficacy of 5%, 10% trisodium citrate and heparin as catheter-locking solution for central venous hemodialysis catheters: a prospective randomized controlled study](https://www.cochranelibrary.com/central/doi/10.1002/central/CN-02202236/full). | Irrelevant to the purpose |
| 73 | [Comparison of the efficacy of gentamicin-citrate and gentamicin-citrate/Taurolock-urokinase locks for non-tunneled catheters in hemodialysis patients](https://www.cochranelibrary.com/central/doi/10.1002/central/CN-02721205/full). | Irrelevant to the purpose |
| 74 | [A Randomized Controlled Clinical Trial of Trisodium Citrate 30% versus Heparin as a Lock Solution for the Prevention of Catheter-Related Infections in Adult Hemodialysis Patients](https://www.cochranelibrary.com/central/doi/10.1002/central/CN-02747456/full). | Irrelevant to the purpose |
| 75 | [Online Simulation-Based Mastery Learning with Deliberate Practice: developing Interprofessional Communication Skill](https://www.cochranelibrary.com/central/doi/10.1002/central/CN-02417308/full). | Irrelevant to the purpose |
| 76 | [Renal perfusion with venous blood extends the permissible suprarenal clamp time in abdominal aortic surgery](https://www.cochranelibrary.com/central/doi/10.1002/central/CN-00638700/full). | Irrelevant to the purpose |
| 77 | [Comparison between standard heparin and tinzaparin for haemodialysis catheter lock](https://www.cochranelibrary.com/central/doi/10.1002/central/CN-00735152/full). | Irrelevant to the purpose |
| 78 | [A Multi-Centered, Two-Arm, Randomized Study Comparing the Effects of AAT-023 (Zuragen) Solution, and Heparin on the Incidence of Catheter Related Blood Stream Infections in Tunneled Chronic Central Venous Catheters for Dialysis](https://www.cochranelibrary.com/central/doi/10.1002/central/CN-02014225/full). | Irrelevant to the purpose |
| 79 | [Low concentration trisodium citrate as a non-inferior locking agent for non-tunneled dialysis catheters in the Asian Setting](https://www.cochranelibrary.com/central/doi/10.1002/central/CN-02092708/full). | Irrelevant to the purpose |
| 80 | [Effect of TauroLock in prevention of catheter-related infection](https://www.cochranelibrary.com/central/doi/10.1002/central/CN-02752061/full). | Irrelevant to the purpose |
| 81 | [EFFECT OF TRISODIUM CITRATE 30% VERSUS HEPARIN AS A CATHETER LOCK SOLUTION ON INFLAMMATORY RESPONSE AND DIALYSIS ADEQUACY IN HEMODIALYSIS PATIENTS](https://www.cochranelibrary.com/central/doi/10.1002/central/CN-02614446/full). | Irrelevant to the purpose |
| 82 | [A Multifaceted Program for Improving Quality of Care in ICU](https://www.cochranelibrary.com/central/doi/10.1002/central/CN-02015384/full). | Irrelevant to the purpose |
| 83 | [Hydrodynamic thrombectomy system versus pulse-spray thrombolysis for thrombosed hemodialysis grafts: a multicenter prospective randomized comparison](https://www.cochranelibrary.com/central/doi/10.1002/central/CN-00330554/full). | Irrelevant to the purpose |
| 84 | [Impact of heparin locking frequency on preventing temporary dialysis catheter dysfunction in haemodialysis patients](https://www.cochranelibrary.com/central/doi/10.1002/central/CN-00667277/full). | Irrelevant to the purpose |
| 85 | [Central Venous Catheter Lock Solution](https://www.cochranelibrary.com/central/doi/10.1002/central/CN-01499950/full). | Irrelevant outcome |
| 86 | [Low-molecular-weight heparin to prevent venous thromboembolism in COVID-19 patients : a randomized controlled trial of different doses](https://www.cochranelibrary.com/central/doi/10.1002/central/CN-02169751/full). | Irrelevant to the purpose |
| 87 | [Anticoagulation therapy for the prevention of hemodialysis tunneled cuffed catheters (TCC) thrombosis](https://www.cochranelibrary.com/central/doi/10.1002/central/CN-00612766/full). | Irrelevant to the purpose |
| 88 | [Anticoagulation therapy for the prevention of hemodialysis tunneled cuffed catheters (TCC) thrombosis](https://www.cochranelibrary.com/central/doi/10.1002/central/CN-00585955/full). | Irrelevant to the purpose |
| 89 | [Arterial line versus venous line administration of low molecular weight heparin, enoxaparin for prevention of thrombosis in the extracorporeal blood circuit of patients on haemodialysis or haemodiafiltration: a randomized cross-over trial](https://www.cochranelibrary.com/central/doi/10.1002/central/CN-01177295/full). | Irrelevant to the purpose |
| 90 | [Comparison of Effectiveness and Safety of Three Lock Solutions for Long-Term Central Venous Catheter for Hemodialysis](https://www.cochranelibrary.com/central/doi/10.1002/central/CN-01554089/full). | Irrelevant to the purpose |
| 91 | [Comparison the efficacy of Heparin 10000 and 5000](https://www.cochranelibrary.com/central/doi/10.1002/central/CN-02351594/full). | Irrelevant to the purpose |
| 92 | [Outcomes of the Use of Sodium Bicarbonate (8.4%) Solution as a Catheter Lock Solution to Prevent Hemodialysis Catheter Loss Due to Lumen Clot Formation](https://www.cochranelibrary.com/central/doi/10.1002/central/CN-01662131/full). | Irrelevant to the purpose |
| 93 | [Evaluation of the Efficacy of an inTerdialytic "Ethanol 40% v/v - enoxapaRin 1000 U/mL" Lock solutioN to Prevent Tunnelled Catheter Infections in Chronic Hemodialysis Patients](https://www.cochranelibrary.com/central/doi/10.1002/central/CN-01562675/full). | Irrelevant to the purpose |
| 94 | [Safety and efficacy of regional citrate anticoagulation in sustained lowefficiency dialysis](https://www.cochranelibrary.com/central/doi/10.1002/central/CN-01060951/full). | Irrelevant to the purpose |
| 95 | [Effect of ethanol-sodium citrate solution with heparin for inhibition of related infections of hemodialysis catheters and their dysfunction in patients under chronic hemodialysis](https://www.cochranelibrary.com/central/doi/10.1002/central/CN-02410925/full). | Irrelevant to the purpose |
| 96 | [The efficacy and safety of 7.5% Sodium bicarbonate versus Heparin for Locking Solution of Acute temporal dailysis catheter : the randomized control study](https://www.cochranelibrary.com/central/doi/10.1002/central/CN-02189904/full). | Irrelevant to the purpose |
| 97 | [Maintenance of adequate hemodialysis access. Prevention of neointimal hyperplasia](https://www.cochranelibrary.com/central/doi/10.1002/central/CN-00123126/full). | Irrelevant to the purpose |
| 98 | [Local delivery of heparin with balloon angioplasty: results of a prospective randomized trial](https://www.cochranelibrary.com/central/doi/10.1002/central/CN-00134599/full). | Irrelevant to the purpose |
| 99 | [Effectiveness of different interdialytic catheter-locking regimens of tunnelled catheters for chronic hemodialysis](https://www.cochranelibrary.com/central/doi/10.1002/central/CN-01172760/full). | Irrelevant to the purpose |
| 100 | [Remifentanil Anesthesia and Postoperative BIS in Cardiac Surgery](https://www.cochranelibrary.com/central/doi/10.1002/central/CN-01505311/full). | Irrelevant to the purpose |
| 101 | [Citrate Anticoagulation vs. Heparin-Coated Dialyzers](https://www.cochranelibrary.com/central/doi/10.1002/central/CN-02013436/full). | Irrelevant to the purpose |
| 102 | Efficacy Study to Evaluate B-Lock" as an Antimicrobial Lock Solution in Dialysis Patients With a CVC. | Irrelevant to the purpose |
| 103 | [Comparison of Effectiveness and Safety of Three Lock Solutions for Long-Term Central Venous Catheter for Hemodialysis](https://clinicaltrials.gov/study/NCT02618317?cond=renal dialysis&term=Central Venous Catheters&intr=heparin-locking solution&rank=2). | Irrelevant to the purpose |
| 104 | Thrombosis-Related Loss of Arterial Lines in the First Wave of COVID-19 and Non-COVID-19 Intensive Care Unit Patients. | Irrelevant to the purpose |
| 105 | Endobronchial Forceps Retrieval of Embedded Inferior Vena Cava Filters: Retrieval of 535 Filters at a Single Center. | Irrelevant to the purpose |
| 106 | A nurse-inserted peripherally inserted central catheter program in general pediatrics: a single-center experience. | Irrelevant to the purpose |
| 107 | Transradial versus transfemoral approach for TACE: a retrospective study. | Irrelevant to the purpose |
| 108 | The effects of pharmacomechanical thrombectomy on novel complete blood count parameters in deep vein thrombosis: A retrospective study. | Irrelevant to the purpose |
| 109 | Improving guideline-concordant thromboprophylaxis prescribing for children admitted to hospital with COVID-19. | Irrelevant to the purpose |
| 110 | Utility and limitations of coherent mapping algorithm utilizing vectors and global propagation patterns in atrial tachycardia. | Irrelevant to the purpose |
| 111 | Phthalide derivative CD21 regulates the platelet- neutrophil extracellular trap-thrombin axis and protects against ischemic brain injury in rodents. | Irrelevant to the purpose |
| 112 | Development of a post-mortem human specimen flow model for advanced bleeding control training. | Irrelevant to the purpose |
| 113 | Peripheral blood stem cells collection by apheresis in very low-weight children with malignant diseases—A single center experience. | Irrelevant to the purpose |
| 114 | Anticoagulant management for transition from failed thrombolysis to extra-corporeal membrane oxygenation in patients with high-risk pulmonary embolism: A thoughtful approach. | Irrelevant to the purpose |
| 115 | CATERPILLAR-study protocol: an assessor-blinded randomised controlled trial comparing taurolidine-citrate-heparin to heparin-only lock solutions for the prevention of central line-associated bloodstream infections in paediatric oncology patients. | Irrelevant to the purpose |
| 116 | SUCCESSFUL THROMBOLYSIS OF AN INTERATRIAL THROMBUS DURING WATCHMAN LAAO. | Irrelevant to the purpose |
| 117 | Iliac Endovascular Intervention Without Procedural Anticoagulation. | Irrelevant to the purpose |
| 118 | Regional citrate anticoagulation vs systemic heparin anticoagulation for double-filtration plasmapheresis. | Irrelevant to the purpose |
| 119 | To learn or not to learn from real-world experience on direct oral anticoagulants in pediatric patients? | Irrelevant to the purpose |
| 120 | The role of stents in hemodialysis vascular access. | Irrelevant to the purpose |
| 121 | The Evaluation of Central Venous Catheter-related Complications in Pediatric Acute Leukemia Patients: Single Center Experience. | Irrelevant to the purpose |
| 122 | Endovascular repair of ruptured external iliac artery pseudoaneurysm and arteriovenous fistula using reversed bell-bottom technique. | Irrelevant to the purpose |
| 123 | Ventricular Septal Rupture - The Resurgence of a Post-Myocardial Infarction Dreadful Complication during COVID-19 Pandemic. | Irrelevant to the purpose |
| 124 | Endocarditis: Who Is Particularly at Risk and Why? Ten Years Analysis of Risk Factors for In-hospital Mortality in Infective Endocarditis. | Irrelevant to the purpose |
| 125 | Perioperative Tracking of Intravenous Iron in Patients Undergoing On-Pump Cardiac Surgery: A Prospective, Single-Center Pilot Trial. | Irrelevant to the purpose |
| 126 | Comparison of Safety of RADial comPRESSion Devices: A Multi-Center Trial of Patent Hemostasis following Percutaneous Coronary Intervention from Conventional Radial Access (RAD-PRESS Trial). | Irrelevant to the purpose |
| 127 | Slow Continuous Ultrafiltration in Regional Citrate Anticoagulation Performed with a Standard Fluid Infusion Central Venous Catheter in Intensive Care Unit for Fluid Overload in Acute on Chronic Heart Failure: A Case Report. | Irrelevant to the purpose |
| 128 | Intravenous dabigatran provides adequate anticoagulation for cardiopulmonary bypass using a rabbit model. | Irrelevant to the purpose |
| 129 | Derivation and Validation of Clinical Phenotypes of the Cardiopulmonary Bypass-Induced Inflammatory Response. | Irrelevant to the purpose |
| 130 | Concomitant transcatheter transfemoral double native valve replacement for severe aortic stenosis and severe mitral stenosis with mitral annular calcification. | Irrelevant to the purpose |
| 131 | RIVAROXABAN FOR THROMBOSIS IN PEDIATRIC PATIENTS: A RETROSPECTIVE OBSERVATIONAL STUDY FROM THE LOW/MIDDLE INCOME SETTING. | Irrelevant to the purpose |
| 132 | Detection of Hyponatremia Development in Hemodialysis Patients by Routine Automated Conductivity-Based Monitoring. | Irrelevant to the purpose |
| 133 | The association between cancer diagnosis, care, and outcomes in 1 million patients hospitalized for acute pulmonary embolism. | Irrelevant to the purpose |
| 134 | Management of Right Atrial Thrombus During MitraClip Implantation: A Case Report and Review of Literature. | Irrelevant to the purpose |
| 135 | Catheter-associated deep vein thrombosis in children with severe traumatic brain injury: A single-center experience. | Irrelevant to the purpose |
| 136 | Single-stage hybrid repair of a right subclavian artery aneurysm involving the origin of the right vertebral artery. | Irrelevant to the purpose |
| 137 | Predictive Value of C2HEST Score for Atrial Fibrillation Recurrence Following Successful Cryoballoon Pulmonary Vein Isolation in Paroxysmal Atrial Fibrillation. | Irrelevant to the purpose |
| 138 | Comparison of spasmolytic regimen for prevention of radial artery spasm during the distal radial approach: A single-center, randomized study. | Irrelevant to the purpose |
| 139 | SAFETY OF DIRECT ORAL ANTICOAGULANTS (DOACS) VS STANDARD OF CARE (SOC) IN CANCER PATIENTS WITH CATHETER ASSOCIATED VENOUS THROMBOEMBOLISM. | Irrelevant to the purpose |
| 140 | RISK FACTORS FOR MISSED DOSES OF PHARMACOLOGIC VENOUS THROMBOEMBOLISM (VTE) PROPHYLAXIS IN TRAUMA PATIENTS: A MULTICENTER PROSPECTIVE STUDY. | Irrelevant to the purpose |
| 141 | Safety and outcome of ultrasound-guided tunneled central venous catheter in children with cancers from low middle-income country: A prospective study. | Irrelevant to the purpose |
| 142 | The Anti-Inflammatory and Antioxidant Effects of Propofol and Sevoflurane in Children With Cyanotic Congenital Heart Disease. | Irrelevant to the purpose |
| 143 | Letter to the Editor: Late Palliative Care Referral: Is Anything Changing? | Irrelevant to the purpose |
| 144 | Prevalence and Severity of Aortic Regurgitation Due to a Percutaneous Left Ventricular Assist Device (Impella 5.0): A Retrospective Observational Study. | Irrelevant to the purpose |
| 145 | Clinical monitoring of activated clotting time during cardiothoracic surgery: comparing the Hemochron(®) Response and Hemochron(®) Signature Elite. | Irrelevant to the purpose |
| 146 | A compartment syndrome complicated by transradial catheterization: a pitfall between guiding catheter and guidewire. | Irrelevant to the purpose |
| 147 | Soft Tissue Reconstruction of Complex Infrainguinal Wounds Following Revisionary Vascular Surgery. | Irrelevant to the purpose |
| 148 | Analysis of the extracorporeal anticoagulation effect of modified citrate infusion during continuous renal replacement therapy in critically ill patients. | Irrelevant to the purpose |
| 149 | Intracardiac Thrombi in Preterm Infants-A Case Study and Review of the Literature. | Irrelevant to the purpose |
| 150 | Venous thromboembolism risk after spinal cord injury: A secondary analysis of the CLOTT study. | Irrelevant to the purpose |
| 151 | Incidence and Risk Factors of Venous Thromboembolism in Childhood Acute Lymphoblastic Leukaemia–a Population-Based Analysis of the Austrian Berlin-Frankfurt-Münster (BFM) Study Group. | Irrelevant to the purpose |
| 152 | Endovascular Lymphatic Decompression via Thoracic Duct Stent Placement for Refractory Ascites in Patients with Cirrhosis: A Pilot Study. | Irrelevant to the purpose |
| 153 | Effect of taurolidine citrate and unfractionated heparin on inflammatory state and dialysis adequacy in hemodialysis patients. | Irrelevant to the purpose |
| 154 | Impact of central venous port implantation method and access choice on outcomes. | Irrelevant to the purpose |
| 155 | Pediatric cancer-associated thrombosis: Analysis from a tertiary care cancer center in India. | Irrelevant to the purpose |
| 156 | Anesthesia Management for the Patient with Chronic Decompensated Heart Failure and Low Cardiac Output Undergoing CABG with Advanced Cardiac Monitoring: A Case Report. | Irrelevant to the purpose |
| 157 | Management of complex juxtarenal total aortoiliac occlusion following failed open and endovascular interventions. | Irrelevant to the purpose |
| 158 | Therapeutic Plasma Exchange: Core Curriculum 2023. | Irrelevant to the purpose |
| 159 | Epicardial access facilitated by carbon dioxide insufflation via intentional coronary vein exit: step-by-step description of the technique and review of the literature. | Irrelevant to the purpose |
| 160 | Subcutaneously anchored securement for peripherally inserted central catheters: Immediate, early, and late complications. | Irrelevant to the purpose |
| 161 | Systematic review of locking solutions for non-tunneled hemodialysis catheters. | Irrelevant to the purpose |
| 162 | Anaesthesia for vascular emergencies – a state of the art review. | Irrelevant to the purpose |
| 163 | In Vivo Effectiveness of Several Antimicrobial Locks To Eradicate Intravascular Catheter Coagulase-Negative Staphylococci Biofilms. | Irrelevant to the purpose |
| 164 | Using Transesophageal Echocardiography in Liver Transplantation with Veno-Venous Bypass Is a Tool with Many Applications: A Case Series from an Italian Transplant Center. | Irrelevant to the purpose |
| 165 | A technique for a single-stage off-pump repair of Kommerell diverticulum with antegrade branch vessel reconstruction. | Irrelevant to the purpose |
| 166 | Cloud-based fusion imaging improves operative metrics during fenestrated endovascular aneurysm repair. | Irrelevant to the purpose |
| 167 | Three-dimensional mapping, recording and ablation in simulated and induced ventricular tachyarrhythmias during mechanical circulatory support using the percutaneous heart pump. | Irrelevant to the purpose |
| 168 | Main operating room deliveries for patients with high-risk cardiovascular disease. | Irrelevant to the purpose |
| 169 | Efficacy of Hemoperfusion in Severe and Critical Cases of COVID-19. | Irrelevant to the purpose |
| 170 | Pilot Randomized Trial of a Three Times Weekly Heparin Flushing Intervention in Children, Adolescents, and Young Adults With Cancer With Tunneled Central Venous Catheters. | Irrelevant to the purpose |
| 171 | The Analgesic Effects of the Addition of Intravenous Ibuprofen to a Multimodal Analgesia Regimen for Pain Management After Pediatric Cardiac Surgery: A Randomized Controlled Study. | Irrelevant to the purpose |
| 172 | Transesophageal Echocardiography-Guided Transseptal Left Atrial Access to Improve Safety in Patients Undergoing Pulmonary Vein Isolation. | Irrelevant to the purpose |
| 173 | Safety and feasibility of a 7F thin-walled sheath via distal transradial artery access for complex coronary intervention. | Irrelevant to the purpose |
| 174 | Comparison of the Effect of Heparinized Normal Saline Solution Versus Saline Solutions in Arterial and Central Venous Catheters on Complete Blood Count After Cardiac Surgery. | Irrelevant to the purpose |
| 175 | Clinical Characteristics of Venous Thrombosis Associated with Peripherally Inserted Central Venous Catheter in Premature Infants. | Irrelevant to the purpose |
| 176 | Impact of Double-Machine Replacement Protocol at Start of Continuous Renal Replacement Therapy in Vasopressor-Dependent Patients: A Retrospective Cohort Study. | Irrelevant to the purpose |
| 177 | Cardiac Tomography and Cardiac Magnetic Resonance to Predict the Absence of Intracardiac Thrombus in Anticoagulated Patients Undergoing Atrial Fibrillation Ablation. | Irrelevant to the purpose |
| 178 | Heparin Low Doses and Standard Doses Effect on Trans-Radial Catheterization. | Irrelevant to the purpose |
| 179 | Incidence of upper extremity deep vein thrombosis in the retrosternal reconstruction after esophagectomy. | Irrelevant to the purpose |
| 180 | The effect of an intermediate dose of Heparin solution in the Prevention of Central Venous Catheter occlusion on ICU admitted patients: A randomized Controlled Trial. | Irrelevant to the purpose |
| 181 | Are the Placement, Maintenance, and Removal of Femoral and Sciatic Catheters Associated with Bleeding Complications in Vascular Patients on Antithrombotics? A Single-Center, Retrospective Cohort Study. | Irrelevant to the purpose |
| 182 | Reevaluation of lock solutions for Central venous catheters in hemodialysis: a narrative review. | Irrelevant to the purpose |
| 183 | Artificial Extracellular Matrix Composed of Heparin-Mimicking Polymers for Efficient Anticoagulation and Promotion of Endothelial Cell Proliferation. | Irrelevant to the purpose |
| 184 | Use of regional citrate anticoagulation with medium cut-off membrane: pilot report. | Irrelevant to the purpose |
| 185 | Meta-analysis of the efficacy of taurolidine in reducing catheter-related bloodstream infections for patients receiving parenteral nutrition. | Irrelevant to the purpose |
| 186 | Analysis of systemic vascular resistance after cardiac surgery: a retrospective cohort study. | Irrelevant to the purpose |
| 187 | Routine Catheter Lock Solutions in Pediatric Cancer Care: A Pilot Randomized Controlled Trial of Heparin vs Saline. | Irrelevant to the purpose |
| 188 | Clot accumulation at the tip of hemodialysis catheters in a large animal model. | Irrelevant to the purpose |
| 189 | Enoxaparin Reduces Catheter-associated Venous Thrombosis After Infant Cardiac Surgery. | Irrelevant to the purpose |
| 190 | A Multi-Centre, Single-Arm Clinical Study to Confirm Safety and Performance of PuraStat (R), for the Management of Bleeding in Elective Carotid Artery Surgery. | Irrelevant to the purpose |
| 191 | Midterm clinical outcome of a case of aortic arch aneurysm treated with a novel arch stentgraft system. | Irrelevant to the purpose |
| 192 | Mid-Term Clinical Outcomes Following Drug-Coated Balloons in Coronary Artery Disease. | Irrelevant to the purpose |
| 193 | UNCONVENTIONAL APPORACH TO DECLOTTING. | Irrelevant to the purpose |
| 194 | Can Right Ventricular Branch Bypass Alleviate Right Ventricular Dysfunction? | Irrelevant to the purpose |
| 195 | Catheter-Directed Thrombolysis vs Anticoagulation in Patients With Acute Intermediate-High-risk Pulmonary Embolism The CANARY Randomized Clinical Trial. | Irrelevant to the purpose |
| 196 | Thromboembolic Events in Patients Undergoing Neoadjuvant Chemotherapy and Radical Cystectomy for Muscle-Invasive Bladder Cancer: A Study of Renal Impairment in Relation to Potential Thromboprophylaxis. | Irrelevant to the purpose |
| 197 | Evaluation of heparinized syringes for measuring newborn metabolites in neonates with a central arterial line. | Irrelevant to the purpose |
| 198 | Transcatheter aortic valve implantation in patients with uninterrupted vitamin K antagonists. | Irrelevant to the purpose |
| 199 | Investigation of Risk Factors for Tunneled Hemodialysis Catheter Dysfunction. | Irrelevant to the purpose |
| 200 | Paediatric Thrombosis: A Five-Year Experience From A Tertiary Care Center Of Pakistan. | Irrelevant to the purpose |
| 201 | Extrinsic Arterial Compression by a Venous Stent Graft in a Patient Undergoing Dialysis. | Irrelevant to the purpose |
| 202 | The Role of Excessive Anticoagulation and Missing Hyperinflammation in ECMO-Associated Bleeding. | Irrelevant to the purpose |
| 203 | Study Protocol: A Randomized Controlled Prospective Single-Center Feasibility Study of Rheopheresis for Raynaud’s Syndrome and Digital Ulcers in Systemic Sclerosis (RHEACT Study). | Irrelevant to the purpose |
| 204 | A novel nonlinear afterload for ex vivo heart evaluation: Porcine experimental results. | Irrelevant to the purpose |
| 205 | Use of Meropenem and Other Antimicrobial Lock Therapy in the Treatment of Catheter-Related Blood Stream Infections in Neonates: A Retrospective Study. | Irrelevant to the purpose |
| 206 | Prophylaxis of Venous Thromboembolism in Children: A Systematic Review. | Irrelevant to the purpose |
| 207 | Evaluation of the incidence of radial artery occlusion using different introducer sheaths and hemostasis techniques. | Irrelevant to the purpose |
| 208 | Heparin Versus Normal Saline: Flushing Effectiveness in Managing Central Venous Catheters in Pediatric Patients With Cancer. | Irrelevant to the purpose |
| 209 | Heparin versus 0.9% sodium chloride intermittent flushing for preventing occlusion in newborns with peripherally inserted central catheters: A systematic review protocol. | Irrelevant to the purpose |
| 210 | Serial Peripheral Nerve Blocks to Aid in Salvage of a Compromised Limb: A Case Report. | Irrelevant to the purpose |
| 211 | Rivaroxaban Reduces the Dabigatran Dose Required for Anticoagulation During Simulated Cardiopulmonary Bypass. | Irrelevant to the purpose |
| 212 | Radiofrequency ablation of atrial fibrillation—50 W or 90 W? | Irrelevant to the purpose |
| 213 | Heparin versus 0.9% sodium chloride locking for prevention of occlusion in central venous catheters in adults. | Irrelevant to the purpose |
| 214 | Characteristics and predictors of venous thrombosis recurrence in patients with cancer and catheter-related thrombosis. | Irrelevant to the purpose |
| 215 | Embolic Protection with the TriGuard 3 System in Nonagenarian Patients Undergoing Transcatheter Aortic Valve Replacement for Severe Aortic Stenosis. | Irrelevant to the purpose |
| 216 | Risk factors for central venous catheter-related thrombosis in hospitalized children: a single-center a retrospective cohort study. | Irrelevant to the purpose |
| 217 | What is known in pre-, peri-, and post-procedural anticoagulation in micro-axial flow pump protected percutaneous coronary intervention? | Irrelevant to the purpose |
| 218 | Systemic Catheter-Related Venous Thromboembolism in Children: Data From the Italian Registry of Pediatric Thrombosis. | Irrelevant to the purpose |
| 219 | Is Deep Vein Thrombosis Chemoprophylaxis Indicated After Spinal Irrigation and Débridement? | Irrelevant to the purpose |
| 220 | Catheter ablation of atrial fibrillation using 2nd-generation cryoballoon in congenital heart disease patients — significance of RF ablation of additional atrial macro-reentrant tachycardia. | Irrelevant to the purpose |
| 221 | Reactivity to heparin in patients with alpha-gal allergy: A potential role for skin testing? | Irrelevant to the purpose |
| 222 | Esophageal image segmentation for guidance of posterior wall lesions during atrial fibrillation ablation. | Irrelevant to the purpose |
| 223 | Endovascular therapies for hepatic artery stenosis post liver transplantation. | Irrelevant to the purpose |
| 224 | Evaluation of the antithrombogenicity of poly-2-methoxyethylacrylate-coated catheters. | Irrelevant to the purpose |
| 225 | Central venous catheter-associated deep vein thrombosis in critically ill pediatric patients: Risk factors, prevention, and treatment. | Irrelevant to the purpose |
| 226 | Visualizable vs. standard, non-visualizable steerable sheath for pulmonary vein isolation procedures: Randomized, single-centre trial. | Irrelevant to the purpose |
| 227 | Implementation of an Evidence-Based Practice Change Removing Heparin From Implanted Vascular Access Devices. | Irrelevant to the purpose |
| 228 | Upper-Extremity Phlegmasia Cerulea Dolens With Compartment Syndrome in Coronavirus Disease 2019 Sepsis. | Irrelevant to the purpose |
| 229 | X-ray exposure in cryoballoon versus radiofrequency ablation for atrial fibrillation over 7 years: A single center study. | Irrelevant to the purpose |
| 230 | Novel intracardiac echocardiography-guided catheter-based removal of inoperable tricuspid valve vegetation. | Irrelevant to the purpose |
| 231 | Effective treatment for massive neonatal catheter-related right atrial thrombosis. | Irrelevant to the purpose |
| 232 | Transradial versus transfemoral arterial access in the uterine artery embolization of fibroids. | Irrelevant to the purpose |
| 233 | Intraarterial papaverine for relief of catheter-induced peripheral arterial vasospasm during pediatric cardiac surgery: A randomized double-blind controlled trial. | Irrelevant to the purpose |
| 234 | Efficacy of systematic catheter locks solution of taurolidine/heparin versus taurolidine/urokinase in end-stage renal insufficiency stage 5D. | Irrelevant to the purpose |
| 235 | A single-center kidney transplantation experience in children with low weight: is low weight a contraindication? | Irrelevant to the purpose |
| 236 | Red blood cell exchange in children with sickle cell disease. | Irrelevant to the purpose |
| 237 | Comparison of chlorhexidine impregnated dressing and standard dressing for the prevention of central-line associated blood stream infection and colonization in critically ill pediatric patients: A randomized controlled trial. | Irrelevant to the purpose |
| 238 | Treatment of Traumatic Internal Carotid Artery Aneurysm by Flow-Diverter: A Single-Center Experience. | Irrelevant to the purpose |
| 239 | Heparin versus 0.9% sodium chloride intermittent flushing for preventing occlusion in newborns with peripherally inserted central catheters: A systematic review protocol. | Irrelevant to the purpose |
| 240 | Angiographic Characteristics of the Vein of Marshall in Patients with and without Atrial Fibrillation. | Irrelevant to the purpose |
| 241 | Risk for excessive anticoagulation during hemodialysis is associated with type of vascular access and bedside coagulation testing: Results of a cross-sectional study. | Irrelevant to the purpose |
| 242 | Symptom Relief and Reintervention after Iliac Vein Stenting Stratified by CEAP Clinical Classification. | Irrelevant to the purpose |
| 243 | Mechanical Thrombectomy via Common Carotid Artery Access in a Neonate with Aortoiliac Occlusion Syndrome. | Irrelevant to the purpose |
| 244 | Prevention of recurrent thrombotic events in children with central venous catheter-associated venous thrombosis. | Irrelevant to the purpose |
| 245 | Evidence on port-locking with heparin versus saline in patients with cancer not receiving chemotherapy: A randomized clinical trial. | Irrelevant to the purpose |
| 246 | Primary Catheter-Directed Thrombolysis for Porto-Mesenteric Venous Thrombosis (PMVT) in Non-Cirrhotic Patients. | Irrelevant to the purpose |
| 247 | Pre-Emptive Antimicrobial Locks Decrease Long-Term Catheter-Related Bloodstream Infections in Hemodialysis Patients. | Irrelevant to the purpose |
| 248 | UTILITY OF RETEPLASE IN MANAGEMENT OF DYSFUNCTIONAL VASCULAR ACCESS IN INDIAN ESRD PATIENTS. | Irrelevant to the purpose |
| 249 | Externalized Reusable Permanent Pacemaker for Prolonged Temporary Cardiac Pacing in Critical Cardiac Care Units: An Observational Monocentric Retrospective Study. | Irrelevant to the purpose |
| 250 | Drug-induced glove and stocking distribution rash: a rare entity in the intensive care unit. | Irrelevant to the purpose |
| 251 | Concentrated sodium citrate catheter lock: "The best laid schemes o' mice an' men…". | Irrelevant to the purpose |
| 252 | Management of antithrombotic treatment and bleeding disorders in patients requiring venous access devices: A systematic review and a GAVeCeLT consensus statement. | Irrelevant to the purpose |
| 253 | Determinants of Arterial Pressure of Oxygen and Carbon Dioxide in Patients Supported by Veno-Arterial ECMO. | Irrelevant to the purpose |
| 254 | Interventions for treating catheter-related bloodstream infections in people receiving maintenance haemodialysis. | Irrelevant to the purpose |
| 255 | Heparin versus 0.9% saline solution to maintain patency of totally implanted venous access ports in cancer patients: A systematic review and meta-analysis. | Irrelevant to the purpose |
| 256 | A Case Series: Continuous Kidney Replacement Therapy in Neonates With Low Body Weight. | Irrelevant to the purpose |
| 257 | A randomized controlled trial of comparative effectiveness between sodium bicarbonate and heparin as a locking solution for tunnelled central venous catheters among haemodialysis patients. | Irrelevant to the purpose |
| 258 | Evaluating the safety and efficacy of argatroban locking solution in the prevention of the dysfunction of haemodialysis central venous catheters: a study protocol for a randomized controlled trial. | Irrelevant to the purpose |
| 259 | Evaluating the safety and efficacy of argatroban locking solution in the prevention of the dysfunction of haemodialysis central venous catheters: a study protocol for a randomized controlled trial. | Irrelevant to the purpose |
| 260 | Use of figure of eight suture for groin closure with no heparin reversal in patients undergoing cryoballoon ablation for atrial fibrillation. | Irrelevant to the purpose |
| 261 | The best solution down the line: an observational study on taurolidine- versus citrate-based lock solutions for central venous catheters in hemodialysis patients. | Irrelevant to the purpose |
| 262 | Prophylactic antibiotics for preventing gram-positive infections associated with long-term central venous catheters in adults and children receiving treatment for cancer. | Irrelevant to the purpose |
| 263 | Aggressive Therapy for Acute Pulmonary Embolism: Systemic Thrombolysis and Catheter-Directed Approaches. | Irrelevant to the purpose |
| 264 | Comparison between the effects of normal saline with and without heparin for the prevention and management of arterial catheter occlusion: a triple-blinded randomized trial. | Irrelevant to the purpose |
| 265 | Locked Away-Prophylaxis and Management of Catheter Related Thrombosis in Hemodialysis. | Irrelevant to the purpose |
| 266 | Multiple arterial and venous thromboembolism in a male patient with hereditary protein C deficiency A case report. | Irrelevant to the purpose |
| 267 | Secondary Anticoagulation Prophylaxis for Catheter-Related Thrombosis in Pediatric Intestinal Failure: Comparison of Short- Vs Long-Term Treatment Protocols. | Irrelevant to the purpose |
| 268 | Outcomes of Catheter-Related Arterial and Venous Thrombosis After Enoxaparin Therapy in Neonates and Infants With Congenital Heart Disease. | Irrelevant to the purpose |
| 269 | Periprocedural Outcomes in Patients on Chronic Anticoagulation Undergoing Fistulograms. | Irrelevant to the purpose |
| 270 | Heparin Versus Normal Saline: Flushing Effectiveness in Managing Central Venous Catheters in Pediatric Oncology Patients. | Irrelevant to the purpose |
| 271 | Expression Profiles of P53, Caspase-3 and Bcl-2 in Patients Undergoing Congenital Heart Corrective surgery: Combined Effects of Anesthesia and Surgery. | Irrelevant to the purpose |
| 272 | Comparison of Vascular Closure Devices vs Manual Compression After Femoral Artery Puncture in Patients on Oral Anticoagulation - Post Hoc Analysis of the ISAR-CLOSURE Trial. | Irrelevant to the purpose |
| 273 | Central catheter-associated deep vein thrombosis in cancer: clinical course, prophylaxis, treatment. | Irrelevant to the purpose |
| 274 | Feasibility of anticoagulation using low molecular-weight heparin during catheter-directed thrombolysis for lower extremity deep venous thrombosis. | Irrelevant to the purpose |
| 275 | Malfunctioning temporary hemodialysis catheters in patients with novel coronavirus disease 2019. | Irrelevant to the purpose |
| 276 | EUROPEAN JOURNAL OF VASCULAR AND ENDOVASCULAR SURGERY. | Irrelevant to the purpose |
| 277 | Wide-ranging clinical spectrum of paradoxical embolism. | Irrelevant to the purpose |
| 278 | Efficacy of 4% tetrasodium ethylenediaminetetraacetic acid (T-EDTA) catheter lock solution in home parenteral nutrition patients: A quality improvement evaluation. | Irrelevant to the purpose |
| 279 | Impact of locking solutions on conditioning biofilm formation in tunnelled haemodialysis catheters and inflammatory response activation. | Irrelevant to the purpose |
| 280 | Catheter lock solutions for reducing catheter-related bloodstream infections in paediatric patients: a network meta-analysis. | Irrelevant to the purpose |
| 281 | Comparing success rates in central venous catheter salvage for catheter-related bloodstream infections in adult patients on home parenteral nutrition: a systematic review and meta-analysis. | Irrelevant to the purpose |
| 282 | Feasibility and safety of catheter-directed thrombolysis via superficial cubital vein for the treatment of acute massive and submassive pulmonary embolism. | Irrelevant to the purpose |
| 283 | Implanted Port Patency: Comparing Heparin and Normal Saline. | Irrelevant to the purpose |
| 284 | Venous thromboembolism associated with central venous catheters in patients with cancer: From pathophysiology to thromboprophylaxis, areas for future studies. | Irrelevant to the purpose |
| 285 | Short Versus Long Timing of Flushing of Totally Implantable Venous Access Devices When Not Used Routinely: A Systematic Review and Meta-analysis. | Irrelevant to the purpose |
| 286 | Complications and management of a long-term pleural access port in a dog with chronic chylothorax associated with lung lobe torsion. | Irrelevant to the purpose |
| 287 | Central venous catheters are an important factor in paediatric thrombosis. | Irrelevant to the purpose |
| 288 | Surface modification strategies for hemodialysis catheters to preventcatheter-relatedinfections: A review. | Irrelevant to the purpose |
| 289 | Transcarotid transcatheter aortic valve implantation with a novel balloon expandable Myval® THV under the local anesthesia. | Irrelevant to the purpose |
| 290 | Clinical outcomes after TAVR with heparin or bivalirudin as periprocedural anticoagulation in patients with and without peripheral arterial disease: Results from the BRAVO-3 randomized trial. | Irrelevant to the purpose |
| 291 | Use of Catheter Lock Solutions in Patients Receiving Home Parenteral Nutrition: A Systematic Review and Individual-Patient Data Meta-Analysis. | Irrelevant to the purpose |
| 292 | Upper extremities deep vein thrombosis and DOAC treatment: A prospective cohort study. | Irrelevant to the purpose |
| 293 | Inferior vena cava syndrome in neonates: An evidence-based systematic review of the literature. | Irrelevant to the purpose |
| 294 | Comparative efficacy and safety of lock solutions for the prevention of catheter-related complications including infectious and bleeding events in adult haemodialysis patients: a systematic review and network meta-analysis. | Irrelevant to the purpose |
| 295 | High Prevalence and Mortality Associated with Upper Extremity Deep Venous Thrombosis in Hospitalized Patients at a Tertiary Care Center. | Irrelevant to the purpose |
| 296 | Antegrade common femoral artery closure device use is associated with decreased complications. | Irrelevant to the purpose |
| 297 | Simple thrombin-based method for eliminating fibrinogen interference in serum protein electrophoresis of haemodialysed patients. | Irrelevant to the purpose |
| 298 | Low molecular weight heparin for prevention of central venous catheter-related thrombosis in children. | Irrelevant to the purpose |
| 299 | Thrombotic risk in children undergoing orthopedic surgery. | Irrelevant to the purpose |
| 300 | A low dose heparinized saline protocol is associated with improved duration of arterial line patency in critically ill COVID-19 patients. | Irrelevant to the purpose |
| 301 | Risk prediction and new prophylaxis strategies for thromboembolism in cancer. | Irrelevant to the purpose |
| 302 | Meta-analysis of bivalirudin versus heparin in transradial coronary interventions. | Irrelevant to the purpose |
| 303 | Massive right atrium thrombus in an extremely-low-birthweight infant. | Irrelevant to the purpose |
| 304 | Assessment of the Antithrombogenicity of a Poly-2-Methoxyethylacrylate-Coated Central Venous Port-Catheter System. | Irrelevant to the purpose |
| 305 | A comparison of coagulation test results from heparinized central venous catheter and venipuncture. | Irrelevant to the purpose |
| 306 | Use of a radiofrequency guidewire to simplify workflow for left atrium access: a case series. | Irrelevant to the purpose |
| 307 | Current approaches in the treatment of catheter-related deep venous thrombosis in children. | Irrelevant to the purpose |
| 308 | Prophylactic anticoagulants for people hospitalised with COVID-19. | Irrelevant to the purpose |
| 309 | The Incidence and Associated Risk Factors in Central Venous Catheter Associated Infectious Complications within the Autologous Stem Cell Transplant Population of Eastern Health. | Irrelevant to the purpose |
| 310 | Dosage of heparin for patency of the totally mplanted central venous catheter in cancer patients. | Irrelevant to the purpose |
| 311 | Athn 15: Characterizing the Real-World Use of Direct Oral Anticoagulants in Pediatric Patients - Interim Analysis. | Irrelevant to the purpose |
| 312 | Comparison of normal saline with heparin solution for flushing central venous catheter in pediatric patients. | Irrelevant to the purpose |
| 313 | Continuous Low-Dose Heparin Infusion for Catheter-Related Thrombosis Prophylaxis in Critically-Ill Children. | Not conducted on adult patients |
| 314 | Normal saline (0.9% sodium chloride) versus heparin intermittent flushing for the prevention of occlusion in long-term central venous catheters in infants and children. | Irrelevant to the purpose |
| 315 | Nonfemoral Arterial Hemostasis Following Percutaneous Intervention Using a Focused Compression Device. | Irrelevant to the purpose |
| 316 | Antistasis Retrograde Flow Vascular Catheter: A Novel Solution to Thrombogenicity: A Computational Fluid Dynamics Study. | Irrelevant to the purpose |
| 317 | Effect of impregnated central venous catheters on thrombosis in paediatric intensive care: Post-hoc analyses of the CATCH trial. | Irrelevant to the purpose |
| 318 | Apixaban for Routine Management of Upper Extremity Deep Venous Thrombosis (ARM-DVT): Methods of a prospective single-arm management study. | Irrelevant to the purpose |
| 319 | Heparin Resistance due to an Acquired Antithrombin Deficiency in a Patient with Sickle Cell Disease during a Pregnancy Complicated by Bilateral Pulmonary Emboli: A Case Report. | Irrelevant to the purpose |
| 320 | Continuous Veno-Venous Hemodialysis Using the Cardio-Renal Pediatric Dialysis Emergency Machine (TM): First Clinical Experiences. | Irrelevant to the purpose |
| 321 | Describing the Point Prevalence and Characteristics of Venous Thromboembolism in Patients with Thrombotic Thrombocytopenic Purpura. | Irrelevant to the purpose |
| 322 | Vascular access practices for therapeutic apheresis: Results of a survey. | Irrelevant to the purpose |
| 323 | Transradial interventions in contemporary vascular surgery practice. | Irrelevant to the purpose |
| 324 | Prophylactic use of the Angel (R) catheter in a patient with paraneoplastic syndrome scheduled for surgical tumor resection. A case report and literature review. | Irrelevant to the purpose |
| 325 | Prophylactic use of the Angel (R) catheter in a patient with paraneoplastic syndrome scheduled for surgical tumor resection. A case report and literature review. | Irrelevant to the purpose |
| 326 | Locking hemodialysis catheters with trimethoprim-ethanol-Ca-EDTA to prevent bloodstream infections: A randomized, evaluator-blinded clinical trial. | Irrelevant to the purpose |
| 327 | Transfusion-Dependent Anemia and Coagulopathy in a Porcine Model of Pediatric Extracorporeal Membrane Oxygenation (ECMO). | Irrelevant to the purpose |
| 328 | The Cost-effectiveness of Antimicrobial Lock Solutions for the Prevention of Central Line-Associated Bloodstream Infections. | Irrelevant to the purpose |
| 329 | Nursing Interventions to Reduce Peripherally Inserted Central Catheter Occlusion for Cancer Patients: A Systematic Review of Literature. | Irrelevant to the purpose |
| 330 | Comparison of normal saline versus heparin flush solutions for maintaining patency of central venous catheter in cancer patients. | Irrelevant to the purpose |
| 331 | Does ultrasound guidance for peripherally inserted central catheter (PICC) insertion reduce the incidence of tip malposition? - a randomized trial. | Irrelevant to the purpose |
| 332 | Tissue plasminogen activator vs heparin for locking central venous catheters between apheresis procedures. | Irrelevant to the purpose |
| 333 | Advanced stage breast cancer is associated with catheter-tip thrombus formation following implantable central venous port placement. | Irrelevant to the purpose |
| 334 | The anticoagulants rivaroxaban and low molecular weight heparin prevent PICC-related upper extremity venous thrombosis in cancer patients. | Irrelevant to the purpose |
| 335 | Stratification of venous thromboembolism risk in burn patients by Caprini score. | Irrelevant to the purpose |
| 336 | Heparin vs. Normal Saline Locking for Prevention of Catheter Occlusion. | Irrelevant to the purpose |
| 337 | Regular Flush-lock is Unnecessary to Maintain Patency of Resting Totally Implantable Venous Access Device. | Irrelevant to the purpose |
| 338 | Evaluation of the Fourth-Generation FloTrac/Vigileo System in Comparison With the Intermittent Bolus Thermodilution Method in Patients Undergoing Cardiac Surgery. | Irrelevant to the purpose |
| 339 | American Society for Parenteral and Enteral Nutrition Guidelines for the Selection and Care of Central Venous Access Devices for Adult Home Parenteral Nutrition Administration. | Irrelevant to the purpose |
| 340 | Hypotension and Transient ST Elevation after Reversal of Heparin with Protamine. | Irrelevant to the purpose |
| 341 | A Retrospective Review of Hospital-Acquired Venous Thromboembolism at a Large Pediatric Tertiary Care Center. | Irrelevant to the purpose |
| 342 | Reducing catheter-associated complications using 4% sodium citrate versus sodium heparin as a catheter lock solution. | Irrelevant to the purpose |
| 343 | What should be the best dialysis catheter lock in critically ill patients?. | Irrelevant to the purpose |
| 345 | VTE Incidence in RRMM Patients Treated with NOVEL Agents: A Monocentric Real Life Experience. | Irrelevant to the purpose |
| 346 | Eight-week interval in flushing and locking port-a-cath in cancer patients: A single-institution experience and systematic review. | Irrelevant to the purpose |
| 347 | Management of RVAD Thrombosis in Biventricular HVAD Supported Patients: Case Series. | Irrelevant to the purpose |
| 348 | Combined hybrid and open approach to repair of an iatrogenic vertebral artery injury. | Irrelevant to the purpose |
| 349 | Comparative efficacy of various antimicrobial lock solutions for preventing catheter-related bloodstream infections: A network meta-analysis of 9099 patients from 52 randomized controlled trials. | Irrelevant to the purpose |
| 350 | Benefits of Impella and Peripheral Veno-Arterial Extra Corporeal Life Support Alliance. | Irrelevant to the purpose |
| 351 | TAUROLIDINE–CITRATE LINE LOCKS PREVENT RECURRENT CENTRAL LINE–ASSOCIATED BLOODSTREAM INFECTION IN PEDIATRIC PATIENTS. | Irrelevant to the purpose |
| 352 | Variability in the management of line-related upper extremity deep vein thrombosis. | Irrelevant to the purpose |
| 353 | Re-evaluating the protective effect of hemodialysis catheter locking solutions in hemodialysis patients. | Irrelevant to the purpose |
| 354 | Pulmonary thromboses in pediatric acute respiratory distress syndrome. | Irrelevant to the purpose |
| 355 | Evaluation of the Activity of Heparin Injected into the Fully Implantable Catheter for Chemotherapy (Portocath) between Two Moments of Use. | Irrelevant to the purpose |
| 356 | Risk factors for complications in cancer patients with totally implantable access ports: A retrospective study and review of the literature. | Irrelevant to the purpose |
| 357 | Advanced mapping strategies for ablation therapy in adults with congenital heart disease. | Irrelevant to the purpose |
| 358 | Intravascular Cooling Catheter-Related Venous Thromboembolism After Hypothermia: A Case Report and Review of the Literature. | Irrelevant to the purpose |
| 359 | Antithrombotic treatment in neonates and children. | Irrelevant to the purpose |
| 360 | Relationship Between Central Venous Catheter Protein Adsorption and Water Infused Surface Protection Mechanisms. | Irrelevant to the purpose |
| 361 | Case with anaphylactic shock induced by heparin-lock flush injection. | Irrelevant to the purpose |
| 362 | Postmarketing experience with Neutrolin® (taurolidine, heparin, calcium citrate) catheter lock solution in hemodialysis patients. | Irrelevant to the purpose |
| 363 | Venous thromboembolism events and prophylaxis in patients with acute myeloid leukemia. | Irrelevant to the purpose |
| 364 | Systematic Review and Meta-Analysis of the Utilization of Ethanol Locks in Pediatric Patients With Intestinal Failure. | Irrelevant to the purpose |
| 365 | Quantification of Lipoteichoic Acid in Hemodialysis Patients With Central Venous Catheters. | Irrelevant to the purpose |
| 367 | Analysis of Patient Characteristics and Risk Factors for Thrombosis After Surgery for Congenital Heart Disease. | Irrelevant to the purpose |
| 368 | Low-dose, short course alteplase treatment of submassive pulmonary embolism: A case series from the National Institutes of Health Clinical Center. | Irrelevant to the purpose |
| 369 | Heparin versus 0.9% sodium chloride locking for prevention of occlusion in central venous catheters in adults. | Irrelevant to the purpose |
| 370 | Intravascular access devices from an interventional radiology perspective: indications, implantation techniques, and optimizing patency. | Irrelevant to the purpose |
| 371 | Heparin Versus Normal Saline: Flushing Effectiveness in Managing Central Venous Catheters in Patients Undergoing Blood and Marrow Transplantation. | Irrelevant to the purpose |
| 372 | Safety and Efficacy of Arterial Closure Devices in an Office-Based Angiosuite. | Irrelevant to the purpose |
| 373 | Establishment of prophylactic enoxaparin dosing recommendations to achieve targeted anti-factor Xa concentrations in children with CHD. | Irrelevant to the purpose |
| 374 | Incidence and outcomes of catheter related thrombosis (CRT) in patients with acute leukemia using a platelet-adjusted low molecular weight heparin regimen. | Irrelevant to the purpose |
| 375 | Direct cost of maintenance of totally implanted central venous catheter patency. | Irrelevant to the purpose |
| 376 | Heparin or Sodium Chloride for Prolonging Peripheral Intravenous Catheter Use in Children - A Systematic Review. | Irrelevant to the purpose |
| 377 | Hemodialysis Catheter-Related Central Venous Thrombosis: Clinical Approach to Evaluation and Management. | Irrelevant to the purpose |
| 378 | Administration of taurolidine-citrate lock solution for prevention of central venous catheter infection in adult neutropenic haematological patients: a randomised, double-blinded, placebo-controlled trial (TAURCAT). | Irrelevant to the purpose |
| 379 | Prevention of hemodialysis catheter infections: Ointments, dressings, locks, and catheter hub devices. | Irrelevant to the purpose |
| 380 | Dialysis catheters in the ICU: selection, insertion and maintenance. | Irrelevant to the purpose |
| 381 | FemoSeal (R) Device Use for Femoral Artery Closure by Different Techniques. | Irrelevant to the purpose |
| 382 | Central Venous Catheter-Associated Deep Venous Thrombosis in Critically Ill Children. | Irrelevant to the purpose |
| 383 | Increased Risk of Thrombosis Associated with Peripherally Inserted Central Catheters Compared with Conventional Central Venous Catheters in Children with Leukemia. | Irrelevant to the purpose |
| 384 | Cluster-Randomized Trial of Devices to Prevent Catheter-Related Bloodstream Infection. | Irrelevant to the purpose |
| 385 | Evaluating the effect of high dose versus low dose heparin in peripherally inserted central catheter in very low birth weight infants. | Not conducted on adult patients. |
| 386 | Convectively Dominated Heparin Leakage From Multiple Catheter Designs: An In Vitro Experimental Study. | Irrelevant to the purpose |
| 387 | Antimicrobial lock solutions for preventing catheter-related infections in haemodialysis. | Irrelevant to the purpose |
| 388 | Safety and efficacy of taurolidine/urokinase versus taurolidine/heparin as a tunneled catheter lock solution in hemodialysis patients: a prospective, randomized, controlled study. | Irrelevant to the purpose |
| 389 | Normal saline versus heparin for patency of central venous catheters in adult patients - a systematic review and meta-analysis. | Irrelevant to the purpose |
| 390 | A comparative study on the use of different connectors in tube sealing in elderly tumor patients with PICC. | Irrelevant to the purpose |
| 391 | Does antimicrobial lock solution reduce catheter-related infections in hemodialysis patients with central venous catheters? A Bayesian network meta-analysis. | Meta-analysis |
| 392 | Necessity of heparin for maintaining peripheral venous catheters: A systematic review and meta-analysis. | Irrelevant to the purpose |
| 393 | Catheter-Directed Thrombolysis in a Child with Bilateral Renal Artery Graft Thrombosis. | Irrelevant to the purpose |
| 394 | Impregnated central venous catheters in children: a systematic review of randomized controlled trials. | Irrelevant to the purpose |
| 395 | Pediatric Hospital Acquired Venous Thromboembolism. | Irrelevant to the purpose |
| 396 | A survey of pediatric hematology/oncology specialists regarding management of central line associated venous thrombosis. | Irrelevant to the purpose |
| 397 | Pharmacomechanical Catheter-Directed Thrombolysis for Deep-Vein Thrombosis. | Irrelevant to the purpose |
| 398 | Taurolidine-citrate-heparin lock reduces catheter-related bloodstream infections in intestinal failure patients dependent on home parenteral support: a randomized, placebo-controlled trial. | Irrelevant to the purpose |
| 399 | Evaluation of a Device Combining an Inferior Vena Cava Filter and a Central Venous Catheter for Preventing Pulmonary Embolism Among Critically III Trauma Patients. | Irrelevant to the purpose |
| 400 | Water Infused Surface Protection as an Active Mechanism for Fibrin Sheath Prevention in Central Venous Catheters. | Irrelevant to the purpose |
| 401 | Port in oncology practice: 3-monthly locking with normal saline for catheter maintenance, a preliminary report. | Irrelevant to the purpose |
| 402 | Ethanol combined with heparin as a locking solution for the prevention of catheter related blood stream infections in hemodialysis patients: A prospective randomized study. | Irrelevant to the purpose |
| 403 | Patterns and Predictors of Peripherally Inserted Central Catheter Occlusion: The 3P-O Study. | Irrelevant to the purpose |
| 404 | Six Weeks Versus 3 Months of Anticoagulant Treatment for Pediatric Central Venous Catheter-related Venous Thromboembolism. | Irrelevant to the purpose |
| 405 | How to perform extracorporeal photopheresis via port catheter. | Irrelevant to the purpose |
| 406 | Vascular access in neonates. | Irrelevant to the purpose |
| 407 | Bivalirudin Versus Unfractionated Heparin During Peripheral Vascular Interventions: A Propensity-matched Study. | Irrelevant to the purpose |
| 408 | Thromboprophylaxis Failure in the Adult Medical Inpatient. | Irrelevant to the purpose |
| 409 | Systematic review of antimicrobial lock therapy for prevention of central-line-associated bloodstream infections in adult and pediatric cancer patients. | Irrelevant to the purpose |
| 410 | A multicenter feasibility study on ultrafiltration via a single peripheral venous access in acute heart failure with overt fluid overload. | Irrelevant to the purpose |
| 411 | Incidence and Determinants of Port Occlusions in Cancer Outpatients A Prospective Cohort Study. | Irrelevant to the purpose |
| 412 | Comparison of linezolid and vancomycin lock solutions with and without heparin against biofilm-producing bacteria. | Irrelevant to the purpose |
| 413 | Deep vein thrombosis in medical and surgical Intensive Care Unit patients in a Tertiary Care Centre in North India: Incidence and risk factors. | Irrelevant to the purpose |
| 414 | Deep vein thrombosis in medical and surgical Intensive Care Unit patients in a Tertiary Care Centre in North India: Incidence and risk factors. | Irrelevant to the purpose |
| 415 | Bivalirudin Versus Heparin for Peripheral Vascular Intervention: You Get What You Pay for. | Irrelevant to the purpose |
| 416 | The impact of clinical practice on the outcome of central venous access devices in children with haemophilia. | Irrelevant to the purpose |
| 417 | High level of venous thromboembolism in critically ill trauma patients despite early and well-driven thromboprophylaxis protocol. | Irrelevant to the purpose |
| 418 | Effects of heparin catheter-sealing solution for implantable venous access ports on D-dimer levels in older cancer patients. | Irrelevant to the purpose |
| 419 | Heparin-Induced Thrombocytopenia. | Irrelevant to the purpose |
| 420 | Phase II Trial on Extending the Maintenance Flushing Interval of Implanted Ports. | Irrelevant to the purpose |
| 421 | A Novel Nonantibiotic Nitroglycerin-Based Catheter Lock Solution for Prevention of Intraluminal Central Venous Catheter Infections in Cancer Patients. | Irrelevant to the purpose |
| 422 | Plastic bronchitis: a rare complication of long-term haemodialysis catheter placement in a child. | Irrelevant to the purpose |
| 423 | Non anti-coagulant factors associated with filter life in continuous renal replacement therapy (CRRT): a systematic review and meta-analysis. | Irrelevant to the purpose |
| 424 | Anticoagulants for the prevention and treatment of catheter-related thrombosis in adults and children on parenteral nutrition: A systematic review and critical appraisal. | Irrelevant to the purpose |
| 425 | Comparative effectiveness of 30 % trisodium citrate and heparin lock solution in preventing infection and dysfunction of hemodialysis catheters: a randomized controlled trial (CITRIM trial). | Irrelevant to the purpose |
| 426 | Anticoagulants and antiplatelet agents for preventing central venous haemodialysis catheter malfunction in patients with end-stage kidney disease. | Irrelevant to the purpose |
| 427 | An ethanol/sodium citrate locking solution compared to heparin to prevent hemodialysis catheter-related infections: a randomized pilot study. | Irrelevant to the purpose |
| 428 | Developmental Hemostasis and Management of Central Venous Catheter Thrombosis in Neonates. | Irrelevant to the purpose |
| 429 | Prevention and Treatment of Venous Thromboembolism in Patients with Cancer: Focus on Drug Therapy. | Irrelevant to the purpose |
| 430 | Ex vivo simulation of cardiopulmonary bypass with human blood for hemocompatibility testing. | Irrelevant to the purpose |
| 431 | Thrombosis in the setting of cancer. | Irrelevant to the purpose |
| 432 | A systematic review and meta-analysis of systemic intraoperative anticoagulation during arteriovenous access formation for dialysis. | Irrelevant to the purpose |
| 433 | Evidence-based criteria for the choice and the clinical use of the most appropriate lock solutions for central venous catheters (Excluding dialysis catheters): A GAVeCeLT consensus. | Irrelevant to the purpose |
| 434 | Heparin-free continuous vena-venous hemofiltration as a veno-venous bypass in inferior vena cava reconstruction. | Irrelevant to the purpose |
| 435 | Heparin-bonded expanded polytetrafluorethylene grafts in hemodialysis access. | Irrelevant to the purpose |
| 436 | Venous access for hematopoietic progenitor cell collection: An international survey by the ASFA HPC donor subcommittee. | Irrelevant to the purpose |
| 437 | Chronic Hemodialysis in Small Children. | Irrelevant to the purpose |
| 438 | Comparison of telavancin and vancomycin lock solutions in eradication of biofilm-producing staphylococci and enterococci from central venous catheters. | Irrelevant to the purpose |
| 439 | Outcomes of accelerated catheter-directed thrombolysis in patients with acute arterial thrombosis. | Irrelevant to the purpose |
| 440 | Anticoagulant effect of low molecular weight heparin on central venous catheters in haemodialysis patients. | Irrelevant to the purpose |
| 441 | Long-Term Radial Artery Patency Following Transradial Coronary Catheterization via a 7-Fr Sheath. | Irrelevant to the purpose |
| 442 | The Influence of Flush Methods on Transfemoral Catheter Cerebral Angiography: Continuous Flush versus Intermittent Flush. | Irrelevant to the purpose |
| 443 | Arterial line versus venous line administration of low molecular weight heparin, enoxaparin for prevention of thrombosis in the extracorporeal blood circuit of patients on haemodialysis or haemodiafiltration: A randomized cross-over trial. | Irrelevant to the purpose |
| 444 | Peripherally inserted central catheter-related vein thrombosis in breast cancer patients. | Irrelevant to the purpose |
| 445 | CATheter Infections in CHildren (CATCH): a randomised controlled trial and economic evaluation comparing impregnated and standard central venous catheters in children. | Irrelevant to the purpose |
| 446 | Low-dose versus high-dose heparin locks for hemodialysis catheters: a systematic review and meta-analysis. | Meta-analysis. |
| 447 | Impregnated central venous catheters for prevention of bloodstream infection in children (the CATCH trial): a randomised controlled trial. | Irrelevant to the purpose |
| 448 | Venous Thromboembolism in Critically Ill Medical Patients Receiving Chemoprophylaxis: A Focus on Obesity and Other Risk Factors. | Irrelevant to the purpose |
| 449 | International clinical practice guidelines including guidance for direct oral anticoagulants in the treatment and prophylaxis of venous thromboembolism in patients with cancer. | Irrelevant to the purpose |
| 450 | Management of catheter-associated upper extremity deep venous thrombosis. | Irrelevant to the purpose |
| 451 | Update on Insertion and Complications of Central Venous Catheters for Hemodialysis. | Irrelevant to the purpose |
| 452 | Heparin versus 0.9% sodium chloride intermittent flushing for the prevention of occlusion in long term central venous catheters in infants and children: A systematic review. | Irrelevant to the purpose |
| 453 | Clinical course of upper extremity deep vein thrombosis in patients with or without cancer: A systematic review. | Irrelevant to the purpose |
| 454 | Strategies to prevent and manage thrombotic complications of acute lymphoblastic leukemia in children and young people vary between centers in the United Kingdom. | Irrelevant to the purpose |
| 455 | Heparinization of long indwelling lines in neonates: Systematic review and practical recommendations. | Irrelevant to the purpose |
| 456 | Case report of a central venous access device-associated thrombosis with aortic embolism in a preterm infant. | Irrelevant to the purpose |
| 457 | The risk of bleeding with tunneled dialysis catheter placement. | Irrelevant to the purpose |
| 458 | Home parenteral nutrition-associated thromboembolic and bleeding events: results of a cohort study of 236 individuals. | Irrelevant to the purpose |
| 459 | Complications associated with central venous access device in children with haemophilia: a nationwide multicentre study in Finland. | Irrelevant to the purpose |
| 460 | Prevention of central venous catheter-related infection in the neonatal unit: a literature review. | Irrelevant to the purpose |
| 461 | Case series of thromboembolic complications in childhood nephrotic syndrome: Hacettepe experience. | Irrelevant to the purpose |
| 462 | Upper limb grafts for hemodialysis access. | Irrelevant to the purpose |
| 463 | Prevention of central venous catheter-associated bloodstream infections in paediatric oncology patients using 70% ethanol locks: A randomised controlled multi-centre trial. | Irrelevant to the purpose |
| 464 | Thrombosis in the Neonatal Intensive Care Unit. | Irrelevant to the purpose |
| 465 | Inhibition of heparin precipitation, bacterial growth, and fungal growth with a combined isopropanol-ethanol locking solution for vascular access devices. | Irrelevant to the purpose |
| 466 | Citrate versus heparin for apheresis catheter locks: An efficacy analysis. | Irrelevant to the purpose |
| 467 | Venous thromboembolism in the ICU: main characteristics, diagnosis and thromboprophylaxis. | Irrelevant to the purpose |
| 468 | Effects of prolonged ethanol lock exposure to carbothane- and silicone-based hemodialysis catheters: a 26-week study. | Irrelevant to the purpose |
| 469 | Arterial line versus venous line administration of lowmolecular weight heparin, enoxaparin for prevention of thrombosis in the extracorporeal blood circuit of patients on haemodialysis or haemodiafiltration-a randomized cross-over trial. | Irrelevant to the purpose |
| 470 | A RIETE registry analysis of recurrent thromboembolism and hemorrhage in patients with catheter-related thrombosis. | Irrelevant to the purpose |
| 471 | Nursing and midwifery practice for maintenance of vascular access device patency. A cross-sectional survey. | Irrelevant to the purpose |
| 472 | Nursing and midwifery practice for maintenance of vascular access device patency. A cross-sectional survey. | Irrelevant to the purpose |
| 473 | Cathasept Line Lock and Microbial Colonization of Tunneled Hemodialysis Catheters: A Multicenter Randomized Controlled Trial. | Irrelevant to the purpose |
| 474 | Central venous catheter-related thrombosis and thromboprophylaxis in children: a systematic review and meta-analysis: discussion. | Irrelevant to the purpose |
| 475 | New technology: heparin and antimicrobial-coated catheters. | Irrelevant to the purpose |
| 476 | Predictors of Venous Thromboembolism Recurrence, Adjusted for Treatments and Interim Exposures: A Population-based Case-cohort Study. | Irrelevant to the purpose |
| 477 | Renal transplantation experience in a patient with factor V Leiden homozygous, MTHFR C677T heterozygous, and PAI heterozygous mutation. | Irrelevant to the purpose |
| 478 | Renal transplantation experience in a patient with factor V Leiden homozygous, MTHFR C677T heterozygous, and PAI heterozygous mutation. | Irrelevant to the purpose |
| 479 | Continuous renal replacement therapy: current practice in Australian and New Zealand intensive care units. | Irrelevant to the purpose |
| 480 | Patterns, risk factors and treatment associated with PICC-DVT in hospitalized adults: A nested case-control study. | Irrelevant to the purpose |
| 481 | Hemodialysis catheter locking solutions and the prevention of catheter dysfunction: a meta-analysis. | Irrelevant to the purpose |
| 482 | Heparin versus 0.9% sodium chloride intermittent flushing for the prevention of occlusion in long term central venous catheters in infants and children. | Irrelevant to the purpose |
| 483 | Venous thromboembolism prophylaxis in critically ill patients. | Irrelevant to the purpose |
| 484 | Concentrated citrate locking in order to reduce the long-term complications of central venous catheters: a randomized controlled trial in patients with hematological malignancies. | Irrelevant to the purpose |
| 485 | Single-Center Experience Comparing the Application of Small-Caliber versus Large-Caliber Arterial Access Closure in a Consecutive Series of Patients. | Irrelevant to the purpose |
| 486 | Convective Leakage Makes Heparin Locking of Central Venous Catheters Ineffective Within Seconds: Experimental Measurements in a Model Superior Vena Cava. | Irrelevant to the purpose |
| 487 | Catheter-associated deep vein thrombosis of the upper extremity in cancer patients: guidance from the SSC of the ISTH. | Irrelevant to the purpose |
| 488 | Citrate Versus Heparin Lock for Hemodialysis Catheters: A Systematic Review and Meta-analysis of Randomized Controlled Trials. | Irrelevant to the purpose |
| 489 | Ethanol versus heparin locks for the prevention of central venous catheter-associated bloodstream infections: a randomized trial in adult haematology patients with Hickman devices. | Irrelevant to the purpose |
| 490 | Thrombolysis for acute deep vein thrombosis. | Irrelevant to the purpose |
| 491 | Risk factors for inpatient venous thromboembolism despite thromboprophylaxis. | Irrelevant to the purpose |
| 492 | Central venous catheter-related thrombosis and thromboprophylaxis in children: A systematic review and meta-analysis. | Irrelevant to the purpose |
| 493 | No impact of endogenous prothrombotic conditions on the risk of central venous line-related thrombotic events in children: results of the KIDCAT study (KIDs with Catheter Associated Thrombosis). | Irrelevant to the purpose |
| 494 | Approach to prophylactic measures for central venous catheter-related infections in hemodialysis: A critical review. | Irrelevant to the purpose |
| 495 | Heparin-induced hyperkalemia in an extremely-low-birth-weight infant: a case report. | Irrelevant to the purpose |
| 496 | Heparin-bonded catheters for prolonging the patency of central venous catheters in children. | Irrelevant to the purpose |
| 497 | Impact of decreased heparin dose for flush-lock of implanted venous access ports in pediatric oncology patients. | Not conducted on adult patients. |
| 498 | Heparin versus normal saline for patency of arterial lines. | Irrelevant to the purpose |
| 499 | A prospective, randomized comparison of three different types of valved and non-valved peripherally inserted central catheters. | Irrelevant to the purpose |
| 500 | Peripherally inserted central venous catheters: frequency of complications in premature newborn depends on the insertion site. | Irrelevant to the purpose |
| 501 | The Use of Vascular Closure Devices and Impact on Major Bleeding and Net Adverse Clinical Events (NACEs) in Balloon Aortic Valvuloplasty: A Sub-Analysis of the BRAVO Study. | Irrelevant to the purpose |
| 502 | Taurolidine lock is superior to heparin lock in the prevention of catheter related bloodstream infections and occlusions. | Irrelevant to the purpose |
| 503 | The authority for certain clinical tasks performed by unlicensed patient care technicians and LPNs/LVNs in the hemodialysis setting: a review. | Irrelevant to the purpose |
| 504 | Effect of port-care frequency on venous port catheter-related complications in cancer patients. | Irrelevant to the purpose |
| 505 | Taurolidine-citrate-heparin catheter lock solution reduces staphylococcal bacteraemia rates in haemodialysis patients. | Irrelevant to the purpose |
| 506 | Comparative effectiveness of two catheter locking solutions to reduce catheter-related bloodstream infection in hemodialysis patients. | Not correlated with the concentration of heparin. |
| 507 | Leakage of central venous catheter locking fluid by hemodynamic transport. | Irrelevant to the purpose |
| 508 | Comparison of ML8-X10 (a prototype oil-in-water micro-emulsion based on a novel free fatty acid), taurolidine/citrate/heparin and vancomycin/heparin antimicrobial lock solutions in the eradication of biofilm-producing staphylococci from central venous catheters. | Irrelevant to the purpose |
| 509 | Targeted temperature management in nursing care. | Irrelevant to the purpose |
| 510 | Heparin versus 0.9% sodium chloride intermittent flushing for prevention of occlusion in central venous catheters in adults. | Irrelevant to the purpose |
| 511 | Management of complications related to central venous catheters in cancer patients: An update. | Irrelevant to the purpose |
| 512 | Endovascular venous thrombolysis in children younger than 24 months. | Irrelevant to the purpose |
| 513 | Efficacy of normal saline in the maintenance of the arterial lines in comparison to heparin flush: a comprehensive review of the literature. | Irrelevant to the purpose |
| 514 | Wandering stent within the pulmonary circulation. | Irrelevant to the purpose |
| 515 | Management of peripherally inserted central catheters (PICC) in pediatric heart failure patients receiving continuous inotropic support. | Irrelevant to the purpose |
| 516 | Venous access catheter-related thrombosis in patients with cancer. | Irrelevant to the purpose |
| 517 | Therapy for cancer-related thromboembolism. | Irrelevant to the purpose |
| 518 | Central vascular catheters and infections. | Irrelevant to the purpose |
| 519 | Flushing the central venous catheter: is heparin necessary? | Irrelevant to the purpose |
| 520 | Activated partial thromboplastin times from venipuncture versus central venous catheter specimens in adults receiving continuous heparin infusions. | Irrelevant to the purpose |
| 521 | Central Venous Catheter Flushing Recommendations: A Systematic Evidence-Based Practice Review. | Irrelevant to the purpose |
| 522 | Concentrated sodium chloride catheter lock solution-a new effective alternative method for hemodialysis patients with high bleeding risk. | Irrelevant to the purpose |
| 523 | Complication profiles of central and non-central 1 Fr PICCs in neonates weighing < 1500 g. | Irrelevant to the purpose |
| 524 | Comparison of heparin to citrate as a catheter locking solution for non-tunneled central venous hemodialysis catheters in patients requiring renal replacement therapy for acute renal failure (VERROU-REA study): study protocol for a randomized controlled trial. | Irrelevant to the purpose |
| 525 | Low molecular weight heparin for prevention of central venous catheterization-related thrombosis in children. | Not conducted on adult patients |
| 526 | The "benefits" of the mini-extracorporeal circulation in the minimal invasive cardiac surgery era. | Irrelevant to the purpose |
| 527 | Coagulation and the surgical neonate. | Irrelevant to the purpose |
| 528 | Heparin bonding does not improve patency of polytetrafluoroethylene arteriovenous grafts. | Irrelevant to the purpose |
| 529 | Continuously intravenous pumping of heparin prevent deep venous thrombosis related with central venous catheter. | Irrelevant to the purpose |
| 530 | Simplified regional citrate anticoagulation using a calcium-containing replacement solution for continuous venovenous hemofiltration. | Irrelevant to the purpose |
| 531 | Sodium citrate 4% versus heparin as a lock solution in hemodialysis patients with central venous catheters. | Irrelevant to the purpose |
| 532 | Peripherally inserted central catheters in infants and children - indications, techniques, complications and clinical recommendations. | Irrelevant to the purpose |
| 533 | Anticoagulant therapies for the prevention of intravascular catheters malfunction in patients undergoing haemodialysis: Systematic review and meta-analysis of randomized, controlled trials. | Irrelevant to the purpose |
| 534 | Heparin does not improve graft function in uncontrolled non-heart-beating lung donation: an experimental study in pigs. | Irrelevant to the purpose |
| 535 | Interventions for restoring patency of occluded central venous catheter lumens (Review). | Irrelevant to the purpose |
| 536 | Prophylactic antibiotics for preventing Gram positive infections associated with long-term central venous catheters in oncology patients. | Irrelevant to the purpose |
| 537 | A National Survey of Neonatal Peripherally Inserted Central Catheter (PICC) Practices. | Irrelevant to the purpose |
| 538 | Ethanol causes protein precipitation - New safety issues for catheter locking techniques. | Irrelevant to the purpose |
| 539 | Central venous catheter care for the patient with cancer: American Society of Clinical Oncology clinical practice guideline. | Irrelevant to the purpose |
| 540 | Subclavian artery pseudoaneurysm complicating central venous catheterization: Endovascular treatment with amplatzer vascular plug 4 and covered stent. | Irrelevant to the purpose |
| 541 | Transovarial venous access in a patient with exhausted vascular access for haemodialysis. | Irrelevant to the purpose |
| 542 | The development of central venous access device flushing guidelines utilizing an evidence-based practice process. | Irrelevant to the purpose |
| 543 | Pros and cons of catheter lock solutions. | Irrelevant to the purpose |
| 544 | Interventional nephrology: Catheter dysfunction- prevention and troubleshooting. | Irrelevant to the purpose |
| 545 | Catheter-related thrombosis in cancer patients. | Irrelevant to the purpose |
| 546 | Upper body central venous catheters in pediatric cardiac surgery. | Irrelevant to the purpose |
| 547 | Low-Dose Heparin Use and the Patency of Peripheral IV Catheters in Children: A Systematic Review. | Irrelevant to the purpose |
| 548 | Heparin infusion through a central line misplaced in the carotid artery leading to hemorrhagic stroke. | Irrelevant to the purpose |
| 549 | Sampling conditions influence multiple electrode platelet aggregometry in cardiac surgery patients. | Irrelevant to the purpose |
| 550 | Variations in the circulating heparin levels during maintenance hemodialysis in patients with end-stage renal disease. | Irrelevant to the purpose |
| 551 | The complete recanalization of PICC-related venous thrombosis in cancer patients: A series of case reports. | Irrelevant to the purpose |
| 552 | Central venous catheters and catheter locks in children with cancer: a prospective randomized trial of taurolidine versus heparin. | Irrelevant to the purpose |
| 553 | Preclosure of large-sized venous access sites in adults undergoing transcatheter structural interventions. | Irrelevant to the purpose |
| 554 | Heparin-binding motifs and biofilm formation by candida albicans. | Irrelevant to the purpose |
| 555 | Impregnated central venous catheters should be readily used to reduce risk of bloodstream infection. | Irrelevant to the purpose |
| 556 | Management of peripherally inserted central catheter associated deep vein thrombosis in children. | Irrelevant to the purpose |
| 557 | Port-a-Cath-related complications in 252 patients with solid tissue tumours and the first report of heparin-induced delayed hypersensitivity after Port-a-Cath heparinisation. | Irrelevant to the purpose |
| 558 | Low-angle vascular access for neurovascular procedures using the arstasis AXERA access device. | Irrelevant to the purpose |
| 559 | Infections increase the risk of central venous catheter-related thrombosis in adult acute myeloid leukemia. | Irrelevant to the purpose |
| 560 | International clinical practice guidelines for the treatment and prophylaxis of thrombosis associated with central venous catheters in patients with cancer. | Irrelevant to the purpose |
| 561 | Cancer-related coagulopathy (Trousseau's syndrome): review of the literature and experience of a single center of internal medicine. | Irrelevant to the purpose |
| 562 | Rescue therapy of difficult-to-treat indwelling central venous catheter-related bacteremias in cancer patients: A review for practical purposes. | Irrelevant to the purpose |
| 563 | Pilot Study Evaluating the Safety of a Combined Central Venous Catheter and Inferior Vena Cava Filter in Critically Ill Patients at High Risk of Pulmonary Embolism. | Irrelevant to the purpose |
| 564 | Stability and compatibility of antimicrobial lock solutions. | Irrelevant to the purpose |
| 565 | Reduction of catheter-related bloodstream infections in preterm infants by the use of catheters with the AgION antimicrobial system. | Irrelevant to the purpose |
| 567 | Activity of ethanol and daptomycin lock on biofilm generated by an in vitro dynamic model using real subcutaneous injection ports. | Irrelevant to the purpose |
| 568 | Cancer patients. | Irrelevant to the purpose |
| 569 | Thrombosis and cancer. | Irrelevant to the purpose |
| 570 | Vascular catheter-related complications in newborns. | Irrelevant to the purpose |
| 571 | Preservative-free 0.9% sodium chloride for flushing and locking peripheral intravenous access device: a prospective controlled trial. | Irrelevant to the purpose |
| 572 | Alteplase for blood flow restoration in hemodialysis catheters: a multicenter, randomized, prospective study comparing "dwell" versus "push" administration. | Irrelevant to the purpose |
| 573 | Venous thromboembolism after trauma: A never event? | Irrelevant to the purpose |
| 574 | Albumin Dialysis Without Anticoagulation in High-Risk Patients: An Observational Study. | Irrelevant to the purpose |
| 575 | Venous thromboembolism in cystic fibrosis. | Irrelevant to the purpose |
| 576 | Pediatric venous thromboembolism in relation to adults. | Irrelevant to the purpose |
| 577 | National survey of central venous catheter flushing in the intensive care unit. | Irrelevant to the purpose |
| 578 | Echocardiographically Detected Fibrinous Sheaths Associated with Central Venous Catheters. | Irrelevant to the purpose |
| 579 | Central venous catheterization and thrombosis in newborns: Update on diagnosis and management. | Irrelevant to the purpose |
| 580 | Heparin or 0.9% sodium chloride to maintain central venous catheter patency: a randomized trial. | Irrelevant to the purpose |
| 581 | Insertion of central venous catheters induces a hypercoagulable state. | Irrelevant to the purpose |
| 582 | Tinzaparin is safe and effective in the management of hemodialysis catheter thrombosis. | Irrelevant to the purpose |
| 583 | Locking Tunneled Hemodialysis Catheters with Hypertonic Saline (26% NaCl) and Heparin to Prevent Catheter-Related Bloodstream Infections and Thrombosis: A Randomized, Prospective Trial. | Irrelevant to the purpose |
| 584 | Catheter Dysfunction: The Role of Lock Solutions. | Irrelevant to the purpose |
| 585 | A randomized trial comparing gentamicin/citrate and heparin locks for central venous catheters in maintenance hemodialysis patients. | Irrelevant to the purpose |
| 586 | Performance comparison of peripherally inserted central venous catheters in gastrointestinal surgery: A randomized controlled trial. | Irrelevant to the purpose |
| 587 | Guidelines for the prevention of intravascular catheter-related infections: Recommendations relevant to interventional radiology for venous catheter placement and maintenance. | Irrelevant to the purpose |
| 588 | Risk factors for upper extremity venous thrombosis associated with peripherally inserted central venous catheters. | Irrelevant to the purpose |
| 589 | Risk factors, management and primary prevention of thrombotic complications related to the use of central venous catheters. | Irrelevant to the purpose |
| 590 | The risk factors and clinical outcomes of upper extremity deep vein thrombosis. | Irrelevant to the purpose |
| 591 | Epidemiology and prevention of catheter-related thrombosis in patients with cancer. | Irrelevant to the purpose |
| 592 | Excimer laser with adjunctive balloon angioplasty and heparin-coated self-expanding stent grafts for the treatment of femoropopliteal artery in-stent restenosis: Twelve-Month Results From the SALVAGE Study. | Irrelevant to the purpose |
| 593 | Catheter-related thrombosis: lifeline or a pain in the neck? | Irrelevant to the purpose |
| 594 | Effect of heparin and other factors associated with complications of peripherally inserted central venous catheters in neonates. | Irrelevant to the purpose |
| 595 | Sodium citrate versus saline catheter locks for non-tunneled hemodialysis central venous catheters in critically ill adults: a randomized controlled trial. | Irrelevant to the purpose |
| 596 | The role of clinically significant venous thromboembolism and thromboprophylaxis in pediatric patients with pelvic or femoral fractures. | Irrelevant to the purpose |
| 597 | Identification of risk factors for catheter-related thrombosis in patients with totally implantable venous access ports in the forearm. | Irrelevant to the purpose |
| 598 | Trisodium citrate 46.7% versus heparin catheter locks for tunneled central venous catheters: A single-center study. | Irrelevant to the purpose |
| 599 | Randomized controlled trial of taurolidine citrate versus heparin as catheter lock solution in paediatric patients with haematological malignancies. | Irrelevant to the purpose |
| 600 | Between Scylla and Charybdis: Antithrombotic therapy in hematopoietic progenitor cell transplant patients. | Irrelevant to the purpose |
| 601 | Significant reduction in central venous catheter-related bloodstream infections in children on HPN after starting treatment with taurolidine line lock. | Irrelevant to the purpose |
| 602 | Efficacy of normal saline versus heparinized saline solution for locking catheters of totally implantable long-term central vascular access devices in adult cancer patients. | Irrelevant to the purpose |
| 603 | Central venous catheter sampling of low molecular heparin levels: an approach to increasing result reliability. | Irrelevant to the purpose |
| 604 | Quantification of systemic delivery of substrates for intermediate metabolism during citrate anticoagulation of continuous renal replacement therapy. | Irrelevant to the purpose |
| 605 | Efficacy of tigecycline and vancomycin in experimental catheter-related Staphylococcus epidermidis infection: microbiological and electron microscopic analysis of biofilm. | Irrelevant to the purpose |
| 606 | Venous thromboembolism in pediatric patients: Epidemiologic data from a pediatric tertiary care center in Alabama. | Irrelevant to the purpose |
| 607 | Efficacy and safety of enoxaparin during hemodialysis: results from the HENOX study. | Irrelevant to the purpose |
| 608 | Complications Associated with 2 Different Types of Percutaneously Inserted Central Venous Catheters in Very Low Birth Weight Infants. | Irrelevant to the purpose |
| 609 | Anticoagulation in the management of venous thromboembolism in the cancer patient. | Irrelevant to the purpose |
| 610 | Hydrophilic surface coatings with embedded biocidal silver nanoparticles and sodium heparin for central venous catheters. | Irrelevant to the purpose |
| 611 | Endoluminal colonization as a risk factor for coagulase-negative staphylococcal catheter-related bloodstream infections in haemodialysis patients. | Irrelevant to the purpose |
| 612 | Diagnosis and management of central-line-associated thrombosis in newborns and infants. | Irrelevant to the purpose |
| 613 | Successful prevention of tunneled, central catheter infection by antibiotic lock therapy using cefotaxime. | Irrelevant to the purpose |
| 614 | Accuracy of bacterial DNA testing for central venous catheter-associated bloodstream infection in children with cancer. | Irrelevant to the purpose |
| 615 | Risk, Clinical Features, and Outcomes of Thrombosis Associated With Pediatric Cardiac Surgery. | Irrelevant to the purpose |
| 616 | A novel antimicrobial and antithrombotic lock solution for hemodialysis catheters: A multi-center, controlled, randomized trial. | Irrelevant to the purpose |
| 617 | Upper-extremity deep venous thrombosis: A review. | Irrelevant to the purpose |
| 618 | Anticoagulation therapy: indications, monitoring, and complications. | Irrelevant to the purpose |
| 619 | Standardized Endpoint Definitions for Transcatheter Aortic Valve Implantation Clinical Trials A Consensus Report From the Valve Academic Research Consortium. | Irrelevant to the purpose |
| 620 | Deep-vein thrombosis of the upper extremities. | Irrelevant to the purpose |
| 621 | Active or passive bio-coating: does it matters in extracorporeal circulation? | Irrelevant to the purpose |
| 622 | Prevention of Dialysis Catheter Malfunction with Recombinant Tissue Plasminogen Activator. | Irrelevant to the purpose |
| 623 | Integrating Evidence-Based Medicine Into the Perioperative Care of Cardiac Surgery Patients. | Irrelevant to the purpose |
| 624 | Internal jugular vein thrombosis: outcome and risk factors. | Irrelevant to the purpose |
| 625 | Thromboembolism in Inflammatory Bowel Disease: An Insidious Association Requiring a High Degree of Vigilance. | Irrelevant to the purpose |
| 626 | How to improve the implementation of guidelines on cancer-related thrombosis. | Irrelevant to the purpose |
| 627 | Upper extremity deep venous thrombosis. | Irrelevant to the purpose |
| 628 | A multicenter survey of heparin prophylaxis practice in pediatric critical care. | Irrelevant to the purpose |
| 629 | Randomized Study of Minocycline and Edetic Acid as a Locking Solution for Central Line (Port-A-Cath) in Children with Cancer. | Irrelevant to the purpose |
| 630 | Preventing venous thrombosis in critically ill children: What is the right approach? | Irrelevant to the purpose |
| 640 | New possibilities of prevention of infection in the newborn. | Irrelevant to the purpose |
| 641 | Catheter Management in Hemodialysis Patients: Delivering Adequate Flow. | Irrelevant to the purpose |
| 642 | Anticoagulation for patients with cancer and central venous catheters. | Irrelevant to the purpose |
| 643 | Central venous catheter-associated thrombosis in the perioperative period: a frequent complication in cancer patients that can be detected early with doppler examination. | Irrelevant to the purpose |
| 644 | A thrombus in the venous reservoir while using bivalirudin in a patient with heparin-induced thrombocytopenia undergoing heart transplantation. | Irrelevant to the purpose |
| 645 | Variations in the application of various perfusion technologies in Great Britain and Ireland - A national survey. | Irrelevant to the purpose |
| 646 | The effect of low-dose heparin on maintaining peripherally inserted percutaneous central venous catheters in neonates. | Not conducted on adult patients. |
| 647 | Patency of arteriovenous fistula for dialysis improbe with topical spraygel heparin. | Irrelevant to the purpose |
| 648 | Antibiotic-based catheter lock solutions for prevention of catheter-related bloodstream infection: a systematic review of randomised controlled trials. | Irrelevant to the purpose |
| 649 | Hemorrhagic shock 3 days after catheterization from the axillary vein. | Irrelevant to the purpose |
| 650 | Heparin or 0.9% sodium chloride flush to maintain central venous catheter patency: A randomized trial. | Irrelevant to the purpose |
| 651 | Traditional and non-traditional strategies to optimize catheter function: Go with more flow. | Irrelevant to the purpose |
| 652 | Editorial: Prevention of catheter-related thrombosis after cardiac surgery: Is heparin the answer? | Irrelevant to the purpose |
| 653 | Upper extremity deep vein thrombosis. | Irrelevant to the purpose |
| 654 | Comparison between standard heparin and tinzaparin for haemodialysis catheter lock. | Not correlated with the concentration of heparin. |
| 655 | Preliminary results of a phase i trial of prophylactic ethanol-lock administration to prevent mediport catheter-related bloodstream infections. | Irrelevant to the purpose |
| 656 | Does low-dose heparin maintain central venous access device patency? A comparison of heparin versus saline during a period of heparin shortage. | Irrelevant to the purpose |
| 657 | Risk Factors of Catheter-related Bloodstream Infection With Percutaneously Inserted Central Venous Catheters in Very Low Birth Weight Infants: A Center's Experience in Taiwan. | Irrelevant to the purpose |
| 658 | Closure Device or Manual Compression in Patients Undergoing Percutaneous Coronary Intervention: A Randomized Comparison. | Irrelevant to the purpose |
| 659 | Hyaluronan based heparin free coated open and closed extracorporeal circuits for high risk coronary revascularization. | Irrelevant to the purpose |
| 660 | Endovascular repair of blunt traumatic thoracic aortic injuries seven-year single-center experience. | Irrelevant to the purpose |
| 661 | Superior vena cava syndrome due to thrombotic occlusion in a thrombophilic renal transplant recipient: a case report. | Irrelevant to the purpose |
| 662 | Heparin-induced thrombocytopenia (HIT) induced by heparin-bonded central venous reservoir placement: A case report. | Irrelevant to the purpose |
| 663 | Diagnosis and initial treatment of venous thromboembolism in patients with cancer. | Irrelevant to the purpose |
| 664 | Cancer-associated thrombosis. | Irrelevant to the purpose |
| 665 | Catheter-associated thrombosis in patients with malignancy. | Irrelevant to the purpose |
| 666 | Totally implantable subpectoral vs. subcutaneous port systems in children with malignant diseases. | Irrelevant to the purpose |
| 667 | Right atrial thrombus due to internal jugular vein catheter. | Irrelevant to the purpose |
| 668 | Sodium citrate versus heparin catheter locks for cuffed central venous catheters: a single-center randomized controlled trial. | Irrelevant to the purpose |
| 669 | Heparin flushing and other interventions to maintain patency of central venous catheters: a systematic review. | Irrelevant to the purpose |
| 670 | Clinical pearls in thrombosis and anticoagulation. | Irrelevant to the purpose |
| 671 | Symptomatic venous thromboembolism: incidence and risk factors in patients with spontaneous or traumatic intracranial hemorrhage. | Irrelevant to the purpose |
| 672 | Venous thromboembolism prophylaxis and treatment in cancer: A consensus statement of major guidelines panels and call to action. | Irrelevant to the purpose |
| 673 | Role of antibiotic lock therapy for the treatment of catheter-related bloodstream infections. | Irrelevant to the purpose |
| 674 | 2008 SOR guidelines for the prevention and treatment of thrombosis associated with central venous catheters in patients with cancer: report from the working group. | Irrelevant to the purpose |
| 675 | Prospective, randomized trial of two different modalities of flushing central venous catheters in pediatric patients with cancer. | Irrelevant to the purpose |
| 676 | Deep vein thrombosis in orthopedic surgery. | Irrelevant to the purpose |
| 677 |  | Irrelevant to the purpose |
| 678 | Catheter-related right atrial thrombus and pulmonary embolism: A case report and systematic review of the literature. | Irrelevant to the purpose |
| 679 | Activity of novel antibiotic lock solutions in a model against isolates of catheter-related bloodstream infections. | Irrelevant to the purpose |
| 680 | Management of occlusion and thrombosis associated with long-term indwelling central venous catheters. | Irrelevant to the purpose |
| 681 | Heparin-bonded central venous catheters do not reduce thrombosis in infants with congenital heart disease: A blinded randomized, controlled trial. | Irrelevant to the purpose |
| 682 | Identifying complications of central venous catheters: infection, thrombosis, occlusion. | Irrelevant to the purpose |
| 683 | Acute promyelocytic leukaemia and acquired alpha-2-plasmin inhibitor deficiency: a retrospective look at the use of epsilon-aminocaproic acid (Amicar) in 30 patients. | Irrelevant to the purpose |
| 684 | Central venous lines for chronic hemodialysis: Survey of the Midwest Pediatric Nephrology Consortium. | Irrelevant to the purpose |
| 685 | Does hemodilution by the crystalloid priming solution derange the efficacy of anticoagulation during cardiopulmonary bypass? | Irrelevant to the purpose |
| 686 | Prevention and management of central venous catheter occlusion and thrombosis in children with cancer. | Irrelevant to the purpose |
| 687 | Taurolidine-citrate lock solution (TauroLock) significantly reduces CVAD-associated grampositive infections in pediatric cancer patients. | Irrelevant to the purpose |
| 688 | Current practice perspectives on the management of thrombosis in children with renal insufficiency: The results of a survey of pediatric hematologists in North America. | Irrelevant to the purpose |
| 689 | Infectious port complications are more frequent in younger patients with hematologic malignancies than in solid tumor patients. | Irrelevant to the purpose |
| 690 | The effect of sodium citrate 4% locking solution for central venous dialysis catheter on the international normalized ratio (INR) value. | Irrelevant to the purpose |
| 691 | A systematic review comparing the relative effectiveness of antimicrobial-coated catheters in intensive care units. | Irrelevant to the purpose |
| 692 | Adjuvant perioperative portal vein or peripheral intravenous chemotherapy for potentially curative colorectal cancer: Long-term results of a randomized controlled trial. | Irrelevant to the purpose |
| 693 | Urokinase lock or flush solution for prevention of bloodstream infections associated with central venous catheters for chemotherapy: A meta-analysis of prospective randomized trials. | Irrelevant to the purpose |
| 694 | A meta-analysis of hemodialysis catheter locking solutions in the prevention of catheter-related infection. | Irrelevant to the purpose |
| 695 | Effectiveness of impregnated central venous catheters for catheter related blood stream infection: a systematic review. | Irrelevant to the purpose |
| 696 | A randomized comparative crossover study to assess the affect on circuit life of varying pre-dilution volume associated with CVVH and CVVHDF. | Irrelevant to the purpose |
| 697 | Thrombotic and infectious complications of central venous catheters in patients with hematological malignancies. | Irrelevant to the purpose |
| 698 | Sublingual administration of warfarin: A novel form of delivery. | Irrelevant to the purpose |
| 699 | Neonatal arterial thrombosis at birth: Case report and literature review. | Irrelevant to the purpose |
| 700 | A randomized, controlled trial of heparin versus placebo infusion to prolong the usability of peripherally placed percutaneous central venous catheters (PCVCs) in neonates: the HIP (Heparin Infusion for PCVC) study. | Irrelevant to the purpose |
| 701 | Increasing AV fistulae and decreasing dialysis catheters: Two aspects of improving patient outcomes. | Irrelevant to the purpose |
| 702 | In vitro activity of daptomycin and vancomycin lock solutions on staphylococcal biofilms in a central venous catheter model. | Irrelevant to the purpose |
| 703 | Antibiotic-lock therapy for long-term catheter-related bacteremia: A review of the current evidence. | Irrelevant to the purpose |
| 704 | Prevention of central venous catheter-associated thrombosis: a meta-analysis. | Meta-analysis |
| 705 | Patency of the ductus arteriosus in the newborn - Now you want it, now you don't. | Irrelevant to the purpose |
| 706 | Anticoagulation for thrombosis prophylaxis in cancer patients with central venous catheters. | Irrelevant to the purpose |
| 707 | Comparison of normal or heparinised saline flushing on function of arterial lines. | Irrelevant to the purpose |
| 708 | Antibiotic lock technique to reduce central venous catheter-related bacteremia. | Irrelevant to the purpose |
| 709 | Activation of coagulation during hemodialysis: effect of blood lines alone and whole extracorporeal circuit. | Irrelevant to the purpose |
| 710 | Peripherally inserted central catheters in children: A survey of practice patterns. | Irrelevant to the purpose |
| 711 | Effect of heparin in arterial line flushing solutions on platelet count: a randomised double-blind study. | Not venous catheters |
| 712 | Catheter connection. | Irrelevant to the purpose |
| 713 | Heparin sensitivity test for patients requiring cardiopulmonary bypass. | Irrelevant to the purpose |
| 714 | The role of low-molecular-weight heparins as supportive care therapy in cancer-associated thrombosis. | Irrelevant to the purpose |
| 715 | Sampling for international normalized ratios in patients on hemodialysis with central venous catheters. | Irrelevant to the purpose |
| 716 | Use of a soft reservoir bag in a fully heparin-coated closed-loop cardiopulmonary bypass system for distal aortic perfusion during aortic surgery. | Irrelevant to the purpose |
| 717 | Reliability of nesiritide infusion via non-primed tubing and heparin-coated catheters. | Irrelevant to the purpose |
| 718 | A vancomycin-heparin lock solution for prevention of nosocomial bloodstream infection in critically ill neonates with peripherally inserted central venous catheters: a prospective, randomized trial. | Irrelevant to the purpose |
